# Adult‐onset idiopathic dystonia: A national data‐linkage study to determine epidemiological, social deprivation, and mortality characteristics

**DOI:** 10.1111/ene.15114

**Published:** 2021-10-15

**Authors:** Grace A. Bailey, Anna Rawlings, Fatemeh Torabi, Owen Pickrell, Kathryn J. Peall

**Affiliations:** ^1^ Neuroscience and Mental Health Research Institute Cardiff University Cardiff UK; ^2^ Swansea University Medical School Swansea UK; ^3^ Health Data Research UK Swansea UK; ^4^ Department of Neurology Morriston Hospital, Swansea Bay University Health Board Swansea UK

**Keywords:** dystonia, incidence, mortality, prevalence, socioeconomic factors

## Abstract

**Background and purpose:**

Accurate epidemiological information is essential for the improved understanding of dystonia syndromes, as well as better provisioning of clinical services and providing context for diagnostic decision‐making. Here, we determine epidemiological, social deprivation, and mortality characteristics of adult‐onset idiopathic dystonia in the Welsh population.

**Methods:**

A retrospective population‐based cohort study using anonymized electronic health care data in Wales was conducted to identify individuals with dystonia between 1 January 1994 and 31 December 2017. We developed a case‐ascertainment algorithm to determine dystonia incidence and prevalence, as well as characterization of the dystonia cohort, based on social deprivation and mortality.

**Results:**

The case‐ascertainment algorithm (79% sensitivity) identified 54,966 cases; of these cases, 41,660 had adult‐onset idiopathic dystonia (≥20 years). Amongst the adult‐onset form, the median age at diagnosis was 41 years, with males significantly older at time of diagnosis compared to females. Prevalence rates ranged from 0.02% in 1994 to 1.2% in 2017. The average annual incidence was 87.7/100,000/year, increasing from 49.9/100,000/year (1994) to 96.21/100,000/year (2017). In 2017, people with dystonia had a similar life expectancy to the Welsh population.

**Conclusions:**

We have developed a case‐ascertainment algorithm, supported by the introduction of a neurologist‐reviewed validation cohort, providing a platform for future population‐based dystonia studies. We have established robust population‐level prevalence and incidence values for adult‐onset idiopathic forms of dystonia, with this reflecting increasing clinical recognition and identification of causal genes. Underlying causes of death mirrored those of the general population, including circulatory disorders, respiratory disorders, cancers, and dementia.

## Graphical abstract

We have developed and validated a case‐ascertainment algorithm, providing a platform for future population‐based dystonia studies. We demonstrate that prevalence and incidence rates of dystonia are higher than previously estimated, 1220/100,000/year (1.2%) and 96/100,000/year, respectively. We have shown that a diagnosis of dystonia does not appear to have a detrimental impact on socioeconomic status, and there was no evidence of decreased life expectancy.

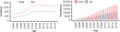

## INTRODUCTION

Dystonia is a hyperkinetic movement disorder characterized by sustained muscle contractions, producing repetitive movements and abnormal postures, impacting both physical and social functioning [1, 2]. Accurate epidemiological information is essential for the improved understanding of dystonia syndromes, as well as better provisioning of clinical services and providing context for diagnostic decision‐making. However, the true prevalence of dystonia remains largely unknown, with notable variation in reported rates worldwide, depending on disorder subtype, cohort ethnicity, and study design [3].

A recent meta‐analysis estimated the global prevalence of dystonia to be 16.43 per 100,000 [4], although work to date suggests that this may vary geographically. Northern European countries, the USA, and Colombia have higher rates of prevalence than those observed in Asia and Southern Europe [5, 6, 7, 8, 9, 10, 11]. Similar variation between ethnicities has also been observed in rates of incidence. Higher rates of adult‐onset idiopathic, isolated focal cervical dystonia (AOIFCD) have been identified amongst Caucasian populations [12, 13]. Higher rates of early‐onset (≤28 years old) idiopathic torsion dystonia (5 per 100,000) were identified amongst Ashkenazi Jewish populations, in part due to the founder effects of genetic forms of dystonia [14]. Prevalence estimates are also dependent on age. The prevalence of early‐onset dystonia (<20 years) is estimated to be between 0.3 and 5 cases per 100,000 [15, 16], whereas the prevalence of adult‐onset (>20 years) dystonia is between 0.3 and 732 cases per 100,000 [3, 15, 17]. Table 1 summarizes dystonia prevalence studies.

**TABLE 1:** Summary of studies investigating the prevalence of dystonia

Author	Year	Country	Study design	Type of dystonia	Age of study population	Population size	Female:male ratio	Mean age at onset, years ± SD	Prevalence per 100,000
Population‐based studies^a^									
Li et al.	1985	China	Population	Focal/generalized	All ages	63,195	‐	‐	Generalized: 5 Focal: 3 Overall: 7.91
Kandil et al.	1994	Egypt	Population	Focal	All ages	42,000	Focal: 0.33:1 Drug‐induced: 1:1 Encephalitic: male Overall: 0.4:1	Focal: 34.6 ± 4.9 Drug‐induced: 28 ± 12.4 Encephalitic: 31.3 ± 6.4	Focal: 10 Drug‐induced: 10 Encephalitic: 7 Overall: 26
Müller et al.	2002	Italy	Population	Focal/segmental	≥50 years	707	1:1	50.5 (median)	732
Jankovic et al.	2007	USA	Population	Focal/segmental	≥18 years	60,062	‐	‐	390
Das et al.	2007	India	Population	Primary	All ages	52,377	‐	‐	Primary: 43.91 Early onset: 5.72 Late onset: 38.18
El Tallawy et al.	2010	Egypt	Population	Not specified	All ages	62,583	‐	‐	30.36
El Tallawy et al.	2013	Egypt	Population	Not specified	All ages	33,285	‐	‐	39.11
Badry et al.	2019	Egypt	Population	Primary	All ages	33,285	2:1	‐	9.01
Service‐based studies^b^									
Nakashima et al.	1995	Japan	Service	Focal	All ages	244,935	1.14:1	‐	Focal: 6.12
ESDE Collaborative Group	2000	France, Austria, England, Spain, Finland, Germany, Portugal, Italy	Service	Focal/segmental	>20 years	5,792,937	Focal: 1.5:1 Segmental: 1.9:1	‐	Focal: 11.7 Segmental: 3.2 Focal and segmental: 14.8
Defazio et al.	2001	Italy	Service	Focal/segmental	All ages	67,606	8:1	Female: 60 Male: 40 Overall: 57.4	Focal and segmental: 13.3 Focal BSP: 7.4 Segmental BSP: 5.9
Castelon Konkiewitz et al.	2002	Germany	Service	Focal/segmental/generalized	All ages	1,322,883	Segmental: 2.3:1 Generalized: 1.0:1	Generalized: 9.8 ± 2.9 Segmental: 55.0 ± 12.8	Focal: 10.1 Segmental: 3.0 Generalized: 0.3 Overall: 17.4
Matsumoto et al.	2003	Japan	Service	Focal/segmental/generalized	All ages	1,459,130	1.1:1	Primary: 48.8 ± 16.5	Generalized: 0.07 Focal: 10.1
Pekmezović et al.	2003	Serbia	Service	Focal/segmental	≥20 years	1,602,226	Focal: 1.5:1 Segmental: 1.1:1 Overall: 1.4:1	Female: 48.8 ± 13.0 Male: 41.8 ± 16.5 Both: 46.0 ± 14.9	Focal: 11.2 Segmental: 2.2 Overall: 13.6
Le et al.	2003	Norway	Service	Focal/segmental	All ages	508,726	2.1:1	46.3	25.4
Sugawara et al.	2006	Japan	Service	Focal/generalized	All ages	1,166,967	‐	‐	Generalized: 0.68 Focal: 14.4 Overall: 15.1
Cossu et al.	2006	Italy	Service	Focal	All ages	1,652,332	2.5:1	58.5 ± 13	3.22
Asgeirsson et al.	2006	Iceland	Service	Focal/segmental/generalized	All ages	288,801	1.9:1	42.7	Generalized: 0.3 Focal: 31.2 Segmental: 3.1 Overall: 37
Fukuda et al.	2006	Japan	Service	Focal	All ages	247,973	0.9:1	Female: 54.3 ± 13.2 Male: 43.5 ± 13.0 Both: 48.6 ± 14.0	13.7
Papantonio et al.	2009	Italy	Service	Focal/segmental	>17 years	541,653	1.46:1	‐	Focal and segmental: 12.74 (adjusted: 13.8)
Bhidayasiri et al.	2011	Thailand	Service	Focal/segmental/generalized	All ages	1,039,595	1.18:1	Males: 42.0 ± 18.1 Females: 47.3 ± 18.4	Primary dystonia: 13.6 Focal dystonia: 14.3 Overall: 19.9
Joensen	2016	Faroe Islands	Service	Focal	All ages	48,100	1.9:1		60.2
Atehortúa et al.	2016	Colombia	Service	Focal/segmental/generalized	All ages	6,221,742	1.6:1	44	Focal: 7.6 Segmental: 1.1 Generalized: 0.7 Overall: 71.2
Wang et al.	2016	China	Service	Late onset	>26 years	54,938,000	2:1	48.54 ± 13.08	2.7
Williams et al.	2017	Ireland	Service	Focal	≥20 years	4,588,252	‐	‐	17.8
Ortiz et al.	2018	Finland	Service	Focal/segmental/generalized	≥20 years	2,043,819	Generalized: 2.5:1 Segmental: 3.7:1 Overall: 2.7:1	Generalized: 49 ± 12 Segmental: 58 ± 12 Overall: 54 ± 12	Generalized: 0.2 Segmental: 3.3 Overall: 40.5
Louis et al.	2019	Faroe Islands	Service	Focal/segmental	≥40 years	24,154	‐		8.28
Record‐linkage studies^c^									
Nutt et al.	1988	USA	Record linkage	Focal/generalized	All ages	56,433	Generalized: male Focal: 1.46:1	Generalized: 20 Focal: ‐	Generalized: 3.4 Focal: 29.5 Overall: 33.67
Duffey et al.	1998	England	Record linkage	Focal/generalized	All ages	2,605,100	Generalized: 1.3:1 Overall: 2.1:1	Generalized: 16.7	Generalized: 1.4 Focal: 12.9 Overall: 14.3
Butler et al.	2004	England	Record linkage	Focal/segmental/generalized	All ages	2,605,100	Generalized: 1.6:1 Focal: 2.2:1 Segmental: 1.9:1	Generalized: 22.2 Focal: 48.3 Segmental: 42.6	Generalized: 3.92 Focal: 38.1 Segmental: 3.99
Hellberg et al.	2019	Sweden	Record linkage	Primary	All ages	9,640,000	‐	‐	35.1

^a^ Population‐based approaches identify cases from the general community to represent the population, for example, door‐to‐door surveys or electronic questionnaires.

^b^ Service‐based studies ascertain cases from inpatient clinics, for example, service users.

^c^ Record‐linkage studies combine data from health and other services, for example, medical records, death records, and socioeconomic status.

Abbreviations: BSP, Blepharospasm.

Different study types have been used to determine the epidemiological characteristics of dystonia. These have included service‐based, linkage record‐based, and population‐based studies. Studies also vary in their reliance upon patient‐reported diagnoses and clinical confirmation (Table 1), possibly explaining the variation in observed prevalence rates. Current estimates of dystonia prevalence are also likely to be underestimates, due to a combination of lack of recognition, underdiagnosis, and the limited number of patients seeking treatment [4, 17].

Recent advances in record‐linked population data repositories have led to increased opportunities for population‐based studies, providing more detailed epidemiological data across a range of neurological disorders [18, 19, 20]. There is a specific need to apply this population‐based approach to dystonia syndromes, particularly in relation to adult‐onset focal syndromes [21]. Use of data from several sources may address conflicting epidemiological estimates, and also has the potential to generate detailed information over several years and across diverse geographic areas, which is currently lacking in the field. The Secure Anonymised Information Linkage (SAIL) Databank contains anonymized, routinely collected data for the Welsh population (3.15 million [2019]) [22]. It is one of the most accessible and broadest sources of anonymized data in the world, covering an extensive percentage of the Welsh population (100% of Welsh hospitals and ~80% of general practitioners). SAIL also provides complete data linkage of social, education, and health care data, enabling a more comprehensive understanding of patient care, and allowing for analysis of multifactorial relationships. In comparison, another UK primary dataset (Clinical Practice Research Datalink) has linkage to only around 10% of English general practices (GPs) [23]. Other linked population data include those in Scandinavia and Australia. However, those in Australia are managed at a state level, including data collections, individual data linkage units, and ethics committees. The Lumos programme, the first state‐wide database linked to GP records throughout New South Wales (NSW), was recently established and covers 16% of the NSW population [24].

Using SAIL, we aimed to derive an algorithm to identify individuals diagnosed with dystonia, and to establish key epidemiological characteristics, including links to deprivation and causes of mortality.

## METHODS

### Study design and data sources

Using a retrospective population‐based cohort study design, we identified patients diagnosed with dystonia within the SAIL Databank (Swansea University, UK: www.saildatabank.com). SAIL is a databank containing anonymized, electronic, person‐based health, social, and education data about the population of Wales. Clinical and demographic data from multiple data‐providing organizations, including Welsh General Practices, Public Health Wales National Health Service (NHS) Trust, Digital Health and Care Wales, HDR Wales, Welsh Government, Office for National Statistics, Welsh Ambulance Service NHS Trust, and Intensive Care National Audit & Research Centre, are anonymized and encrypted by a trusted third party, the NHS Wales Informatics Service, and transferred directly to SAIL. Individuals are reliably matched against records and anonymized using a unique Anonymous Linking Field. This matching algorithm has demonstrated high specificity (>99%) and sensitivity (>95%) and enables linkage across different datasets at a person‐based level [25, 26].

Our study population was formed from primary care (Welsh Longitudinal General Practice dataset) and secondary care (Patient Episode Database for Wales [PEDW] and Outpatient Dataset [OPD]) between January 1994 (utilization of the reference population and the first monogenic form of dystonia identified in 1994 [DYT5a]) [27] and December 2017. At the time of analysis, primary care and secondary care data were available for ~80% and 100% of the Welsh population, respectively. Time coverage varies between GPs, and there are dates with higher periods of data capture within SAIL, for example, 2004–2007. Inpatient (PEDW) and outpatient (OPD) datasets commenced in 1997 and 2004, respectively. We recorded demographic characteristics including age, sex, GP registration history, and deprivation. We measured deprivation using the Welsh Index of Multiple Deprivation (WIMD) obtained from the Welsh Demographic Service Dataset, which contains administrative data on all persons registered with a primary care practice in Wales [28]. Date and cause of death (if applicable) were obtained from the Annual District Death Extract (ADDE). Data were analysed from September 2020 to May 2021.

### Reference population and diagnostic validation

Anonymized records from 90 patients with a confirmed diagnosis of AOIFCD were linked to records held in SAIL. Each participant was recruited via the Welsh Movement Disorders Research Network Study (Research Ethics Committee reference: 14/WA/0017, Integrated Research Application System ID: 146495), was clinically assessed, and had their NHS records reviewed by a neurologist with expertise in movement disorders (K.J.P.). These patients formed the reference population for our derived cohort of individuals diagnosed with dystonia.

### Sensitivity algorithm

A list of Read codes (version 2) and International Classification of Diseases, 10th Revision (ICD‐10) codes was selected and used to identify individuals with a primary and/or secondary dystonia diagnosis. All codes relevant to dystonia including diagnosis, symptoms, and therapy were reviewed by a clinical neurologist with movement disorder expertise. Code lists were created to maximize the positive predictive value, while maintaining a reasonable sensitivity. In a stepwise manner, Read codes that did not contribute to the identification of the reference cohort were removed. All ICD‐10 codes, excluding drug‐induced dystonia, were used. Sensitivity analyses of Read codes are available in Table S1. A full list of the final Read and ICD‐10 codes is provided in Table 2.

**TABLE 2:** ICD‐10 and read codes used to identify dystonia patients in hospital and general practice electronic records, respectively

Dystonia subtype	ICD‐10 code	Read code	Read code description
Genetic torsion dystonia	G24.1		
Idiopathic torsion dystonia		F136.	Idiopathic torsion dystonia
		F137.	Symptomatic torsion dystonia
		F137y	Symptomatic torsion dystonia OS
		F137z	Symptomatic torsion dystonia NOS
		F138.	Fragment of torsion dystonia
		F138z	Torsion dystonia fragment NOS
Idiopathic nonfamilial dystonia	G24.2		
Idiopathic familial dystonia		F1360	Idiopathic familial dystonia
Cervical dystonia	G24.3	F1382	Spasmodic torticollis
		16A3.	Torticollis, symptom
		N135.	Torticollis unspecified
		N1350	Intermittent torticollis
		N135z	Torticollis NOS
Idiopathic Orofacial dystonia	G24.4		
Blepharospasm	G24.5	F1380	Blepharospasm
Writer's cramp		F1383	Organic writer's cramp
Myoclonic dystonia		F13B.	Myoclonic dystonia
Segawa syndrome		F13C.	Segawa syndrome
Other	G24.8	Fyu24	[X]Other dystonia
Unspecified	G24.9	Fyu2A	[X]Dystonia, unspecified
		F13X.	Dystonia, unspecified
Tremor		1B22.	Has a tremor

Abbreviations: [X], external causes of morbidity and mortality; ICD‐10, International Classification of Diseases, 10th Revision; NOS, not otherwise specified; OS, otherwise specified.

### Dystonia diagnosis

An individual was defined as having a diagnosis of dystonia if their GP or hospital record contained a Read version 2 or ICD‐10 code from the list of codes (Table 2). Dystonia subtypes were not mutually exclusive; an individual could have more than one dystonia subtype. Results from primary and secondary care extracts were combined to create a joined dystonia cohort. Individuals diagnosed with a potential secondary cause of dystonia were excluded (Tables [Link], [Link], [Link]) [29, 30]. Our stringent exclusion criteria removed diagnostic codes linked with tremor (apart from dystonic tremor) and diagnoses that may include tremor as part of the phenotype. Medicine codes for all forms of dopaminergic therapy were also included in this exclusion algorithm, providing an additional mechanism to exclude degenerative forms of tremor. Inclusion of the tremor code was shown to increase the sensitivity of our patient identification algorithm by 6% (73%–79%) in our validation cohort, allowing for recognition of dystonic tremor as a form of primary dystonia, while limiting the possibility of including other forms of tremor.

**FIGURE 1:**
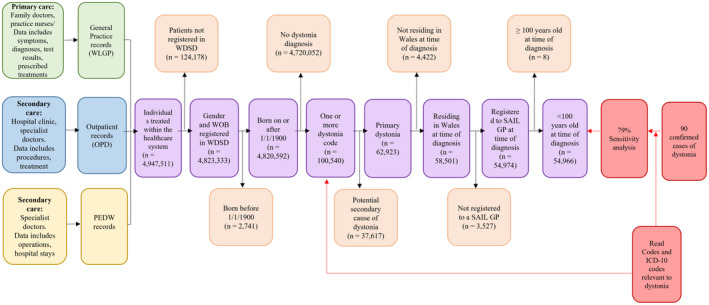
**Flow diagram of cohort development** **.** Green, blue, and yellow boxes: primary and secondary health data records; purple boxes: dystonia cohort population; orange boxes: exclusion criteria; red boxes: sensitivity analysis. GP, general practice, ICD‐10, International Classification of Diseases, 10th Revision; OPD, Outpatient Dataset; PEDW, Patient Episode Database for Wales; SAIL, Secure Anonymised Information Linkage; WDSD, Welsh Demographic Service Dataset; WLGP, Welsh Longitudinal General Practice dataset; WOB, week of birth

Additionally, individuals were required to be resident in Wales at the time of diagnosis and have an age, sex, and GP registration date recorded. Individuals born before 1 January 1900 and older than 100 years were excluded. Entry date into the cohort was the first date of a new dystonia diagnosis and the last date of follow‐up was the earliest GP deregistration (from a contributing practice), date of death, or the end of the study period, whichever was first. Figure 1 summarizes how the dystonia cohort was derived.

We identified all dystonia cases in primary and secondary care datasets; however, further analysis including epidemiological, deprivation, and mortality characterization focuses on individuals with adult‐onset idiopathic dystonia (≥20 years of age).

### Incidence and prevalence

Estimates for prevalence and incidence were calculated annually for the study period (January 1994–December 2017). Prevalence was calculated by dividing the number of people diagnosed with dystonia midyear (30 June) by the midyear population estimate. Incidence was calculated by dividing the number of newly diagnosed cases (no previous diagnosis) for that year by the midyear population estimate. The midyear population was estimated by the total number of Welsh residents with a recorded sex and age, registered to a GP on 30 June of that year.

### Deprivation score

Deprivation scores were derived using WIMD 2014 quintiles according to Lower Super Output Area (LSOA) of residence (2011). WIMD identifies and ranks all LSOAs in Wales from 1 (most deprived) to 1909 (least deprived); each LSOA is then grouped into quintiles, with 1 being the most deprived and 5 being the least deprived [28]. The WIMD quintile was assessed at three dates: study entry, time of dystonia diagnosis, and exit date from the study (study end [December 2017], deregistration from GP, moving out of Wales, or death).

### Mortality

Date and underlying causes of death during the study period were determined from the ADDE. Underlying causes of deaths coded using ICD‐9 were converted to ICD‐10.

### Statistical analysis

Data was analysed using R software (version 4.0.4). Where applicable, a *t*‐test or Mann–Whitney *U*‐test was used to determine any significant difference in the median age at diagnosis and subtype of dystonia, and Wilcoxon signed‐rank test was used to determine changes to WIMD quintile following diagnosis.

### Ethics

This study design uses anonymized, routinely collected data and therefore does not require ethical approval and written informed consent. The SAIL independent Information Governance Review Panel, experts in information governance and members of the public, approved this study (Reference: 0768).

## RESULTS

### Validating the dystonia diagnosis

The optimized case‐ascertainment algorithm (Figure 1) had a diagnostic sensitivity of 79%, identifying 70 of 89 of the clinically confirmed reference population (one patient could not be identified within SAIL).

### Dystonia cohort

A total of 54,966 patients were identified as having been diagnosed with dystonia between 1 January 1994 and 31 December 2017. Of these, 54,488 were identified from primary care (GP records) and 478 from secondary care (hospital records). Of these, 41,660 were determined to have adult‐onset symptoms (age ≥ 20 years) and 13,306 young‐onset forms of dystonia (age <20 years; Table 3). In adult‐onset forms, the female:male ratio was 1.7:1. Fourteen cases had more than one dystonia diagnosis. The most common combination of diagnoses was cervical dystonia and unspecified (*n* = 8). No cases of idiopathic familial dystonia or Segawa syndrome were identified, irrespective of levodopa medication exclusion.

**TABLE 3:** Summary of cohort characteristics for adult‐onset idiopathic cases (≥20 years old)

Characteristic	Overall dystonia	Genetic torsion dystonia	Idiopathic torsion dystonia	Idiopathic nonfamilial dystonia	Cervical dystonia	Idiopathic orofacial dystonia	Blepharospasm	Writer's cramp	Myoclonic dystonia	Other/unspecified	Tremor associated with dystonia
Total participants, *n*	41,660	5	39	<6	26,563	24	1181	41	8	604	13,207
Sex, *n* (%)											
Female	26,311 (63)	^a^	26 (67)	^a^	17,185 (65)	16 (66.7)	773 (65)	25 (61)	^a^	375 (62)	7905 (60)
Male	15,349 (37)	^a^	13 (33)	^a^	9378 (35)	8 (33.3)	408 (35)	16 (39)	^a^	229 (38)	5302 (40)
Median age at diagnosis, years (IQR)	42 (28)	49 (9)	49 (22)	38.5 (13.5)	38 (20)	72.5 (27.25)	48 (29)	40 (21)	44.5 (13.75)	48 (29)	55 (34)
Female median age at diagnosis, years	41^b^	55	47.5	38.5	37^b^	73	51^b^	36	44	48^b^	55
Male median age at diagnosis, years	43	45	50	^d^	39	72.5	45	43.5	68	45	55
Deprivation quintile at diagnosis, *n* (%)											
1: most deprived	7986 (19.17)	^a^	^a^	^a^	4927 (18.55)	^a^	139 (11.77)	6 (14.63)	^a^	134 (22.19)	2768 (20.96)
2	8389 (20.14)	^a^	8 (20.51)	^a^	5294 (19.93)	^a^	218 (18.46)	7 (17.07)	^a^	127 (21.03)	2735 (20.71)
3	8952 (21.49)	^a^	^a^	^a^	5817 (21.90)	^a^	267 (22.61)	10 (24.39)	^a^	119 (19.70)	2731 (20.68)
4	8012 (19.23)	^a^	9 (23.08)	^a^	5169 (19.46)	^a^	258 (21.85)	6 (14.63)	^a^	106 (17.55)	2458 (18.61)
5: least deprived	8321 (19.97)	^a^	13 (33.33)	^a^	5356 (20.16)	^a^	299 (25.32)	12 (29.27)	^a^	118 (19.54)	2515 (19.04)
Median follow‐up, years (IQR)	23.50 (7.65)	9.52 (10.51)	23.98 (8.06)	17.42 (6.60)	23.97 (6.44)	12.46 (8.82)	23.11 (8.40)	21.37 (12.69)	16.76 (7.93)	21.64 (10.54)	22.12 (9.58)
Median change in WIMD quintile at follow‐up (*p*‐value)	0 (<0.001)^c^	0	0 (0.41)^c^	0	0 (<0.001)^c^	0 (0.67)^c^	0 (0.02)^c^	0 (0.34)^c^	0 (1)^c^	0 (0.38)^c^	0 (0.03)^c^
Median change in WIMD quintile from entry into dataset to diagnosis (*p*‐value)	0 (<0.001)^c^	0	0 (0.29)^c^	0	0 (<0.001)^c^	0 (0.37)^c^	0 (<0.01)^c^	0 (0.14)^c^	0 (0.59)^c^	0 (0.57)^c^	0 (<0.001)^c^
Mortality data											
Deceased	4315	<6	5	^d^	1475	13	155	<6	<6	93	2567
Age at death, years											
<45	149	^a^	^a^		71	^d^	^a^	^a^	^a^	6	69
45–64	577	^a^	^a^		253	^d^	^a^	^a^	^a^	12	299
65–84	2048	^a^	^a^		701	^a^	74	^a^	^a^	44	1214
≥85	1541	^a^	^a^		450	^a^	69	^a^	^a^	31	985
Female deceased, *n* (%)	2417 (56)	^a^	^a^		755 (51)	8 (62)	110 (71)	^a^	^a^	64 (69)	1475 (57)
Male deceased, *n* (%)	1898 (44)	^a^	^a^		720 (49)	5 (38)	45 (29)	^a^	^a^	29 (31)	1092 (43)
Median age at death, years (IQR)	80 (17)	65 (10)	70 (7)		79 (19)	76 (12)	83 (13)	70.5 (12.5)	69 (26)	78 (19)	81 (16)
Female median age at death, years	82	65	72.5		80	82.5	85	^a^	^a^	79	83
Male median age at death, years	78	^d^	70		78	75	79	74	^a^	72	78

Note

No cases of Segawa syndrome or idiopathic familial dystonia were identified.

Abbreviations: IQR, interquartile range; WIMD, Welsh Index of Multiple Deprivation.

^a^ Masked to prevent identification of small numbers (<5).

^b^ Significant difference in age at onset between sexes (*p* < 0.05).

^c^ Wilcoxon signed‐rank test.

^d^ No data available.

### Prevalence and incidence rates

**FIGURE 2:**
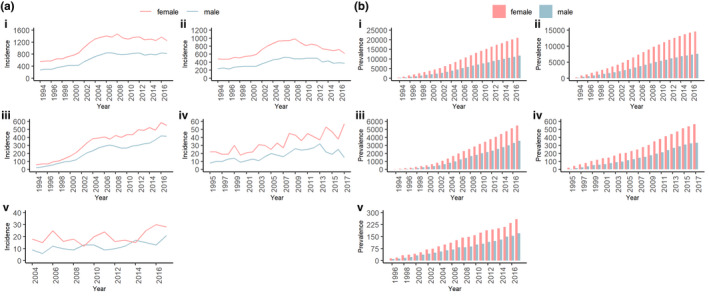
**Trends in (a) incidence and (b) prevalence for adult‐onset idiopathic dystonia by sex and dystonia subtype.** (i) Overall dystonia. (ii) Cervical dystonia. (iii) Tremor associated with dystonia. (iv) Blepharospasm (masked in 1994). (v) Other/unspecified (incidence masked <2004; prevalence masking in 1994 and 1995)

There were 32,662 patients diagnosed with adult‐onset idiopathic dystonia at the midpoint of 2017, providing a crude prevalence of 1.2% amongst the Welsh population. During the study period, 41,660 new cases of adult‐onset idiopathic dystonia were reported, giving a mean incidence of 87.6/100,000/year, varying between 49.9/100,000/year in 1994 and 96.2/100,000/year in 2017. The maximum incidence was 112.7/100,000/year in 2006 (Figure 2a and Table S5). Dystonia incidence increased in males and females, 23.5/100,000 to 62.6/100,000 (1994 and 2017), and 45.5/100,000 to 95.1/100,000 (1994 and 2017), respectively.

### Deprivation

**FIGURE 3:**
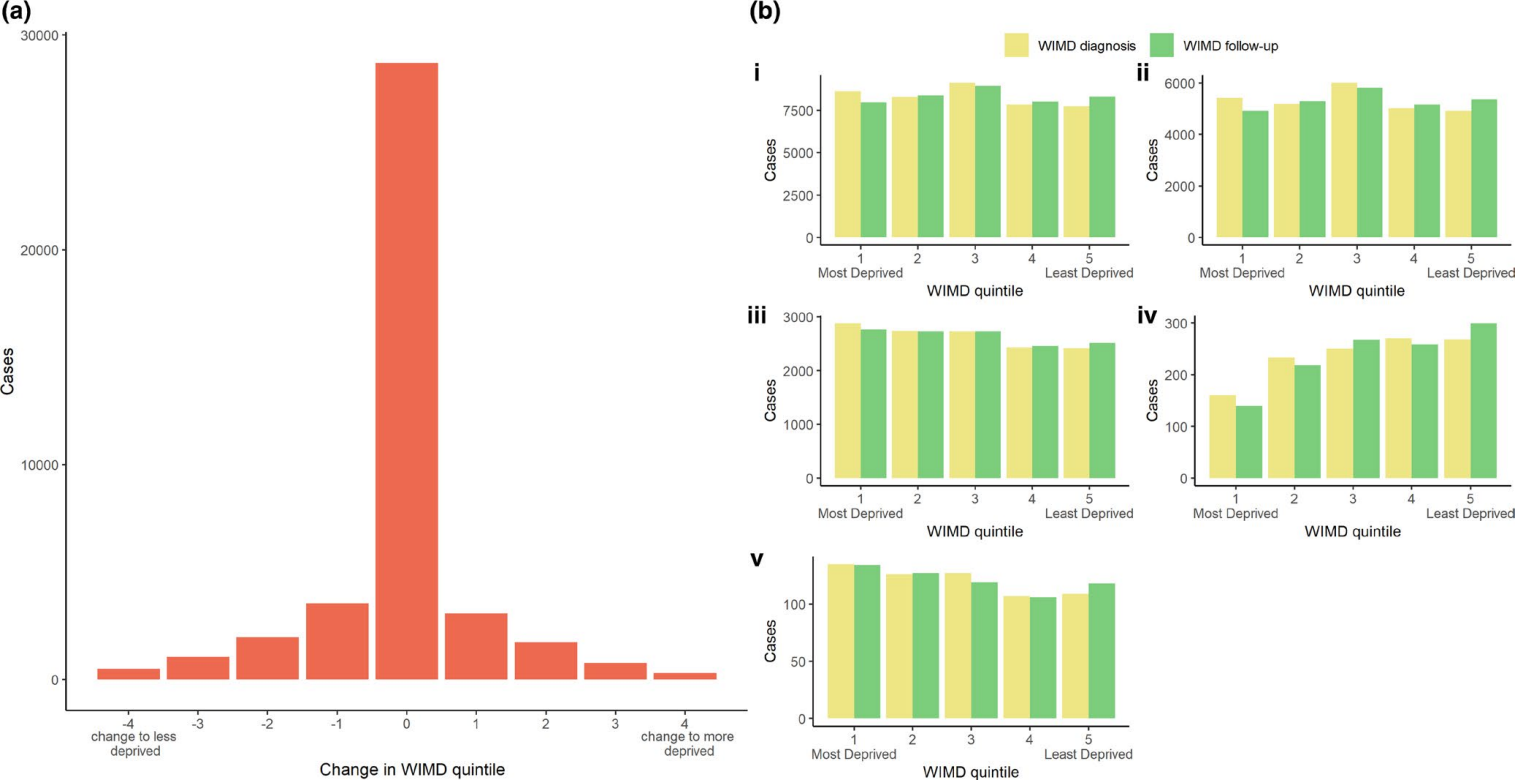
**(a) Change in Welsh Index of Multiple Deprivation (WIMD) quintile and (b) number of cases per WIMD quintile at entry into dataset (during study period) and diagnosis by dystonia subtype in adult‐onset forms.** (i) Overall dystonia. (ii) Cervical dystonia. (iii) Tremor associated with dystonia. (iv) Blepharospasm. (v) Other/unspecified. Change in WIMD quintile = (WIMD quintile at time of diagnosis) – (WIMD quintile at end of follow‐up). WIMD Quintile 1 is most deprived, and Quintile 5 is least deprived

**FIGURE 4:**
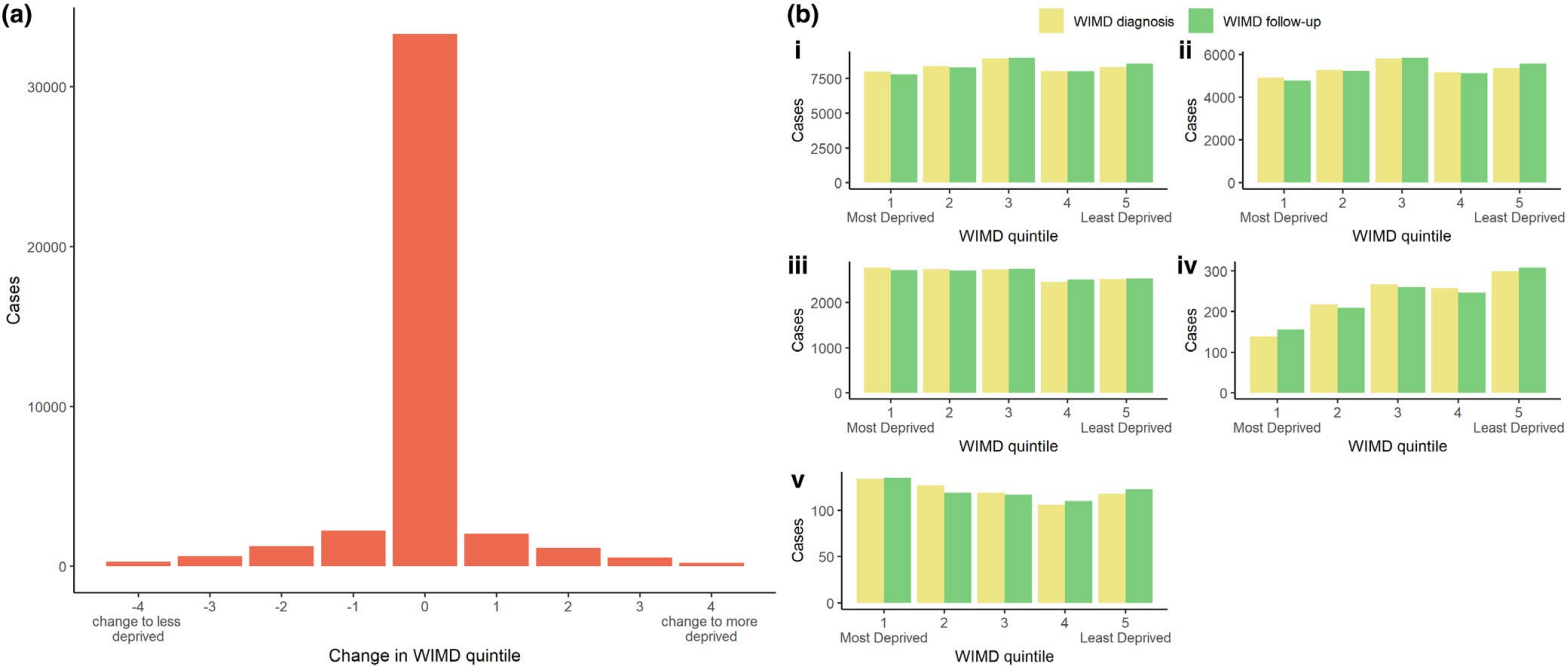
**(a) Change in Welsh Index of Multiple Deprivation (WIMD) quintile and (b) number of cases per WIMD quintile at diagnosis and follow‐up by dystonia subtype in adult‐onset forms. (i) Overall dystonia.** (ii) Cervical dystonia. (iii) Tremor associated with dystonia. (iv) Blepharospasm. (v) Other/unspecified. Change in WIMD quintile = (WIMD quintile at entry) – (WIMD quintile at time of diagnosis). WIMD Quintile 1 is most deprived, and Quintile 5 is least deprived

At the time of diagnosis, adult‐onset cases were equally distributed across deprivation quintiles (Table 3 and Figure 3b). There were no significant changes in deprivation quintile throughout the study between entry into the dataset (during the study period) and diagnosis (median = 0, *p* < 0.01; Figure 3a), diagnosis and follow‐up (median = 0, *p* < 0.01; Figure 4a), and entry into the study and follow‐up (median = 0, *p* < 0.01).

### Mortality

**FIGURE 5:**
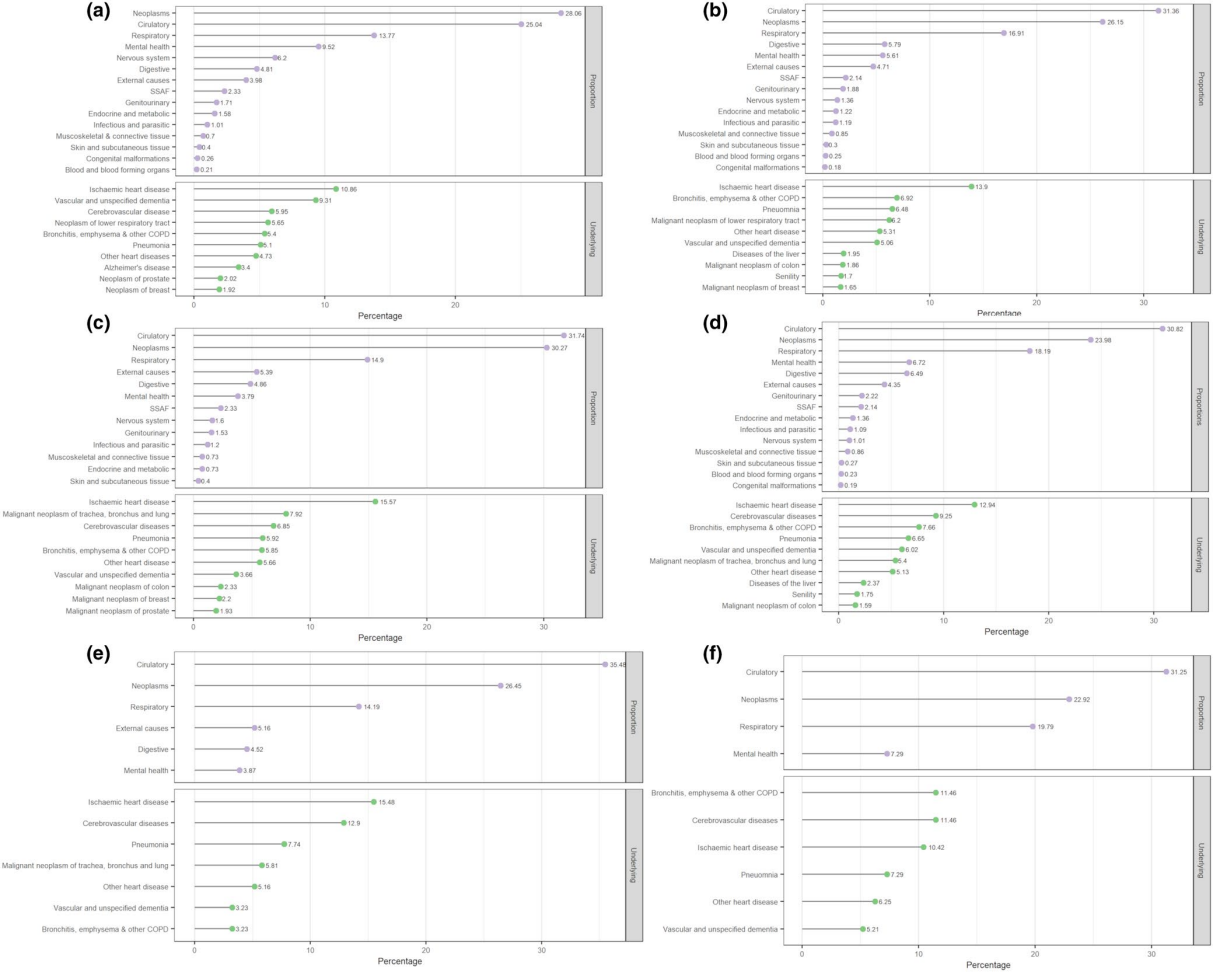
**Mortality data of cases including leading underlying causes of death and proportions of death categorized by causes in adult‐onset idiopathic dystonia.** (a) General population in Wales (2017). (b) Overall dystonia. (c) Cervical dystonia. (d) Tremor associated with dystonia. (e) Blepharospasm. (f) Other/unspecified. COPD, Chronic Obstructive Pulmonary Disease, SSAF, symptoms, signs, and abnormal clinical and laboratory findings, not elsewhere classified

There were 4,315 deaths (10%) in the adult‐onset dystonia cohort (Table 3). Distribution of mortality data by dystonia syndromes, including the 10 most common causes of death, is shown in Figure 5.

## DISCUSSION

Our longitudinal, nationwide population‐based cohort study has combined anonymized electronic data derived from GP (family doctor/community health care) and hospital records for more than 3.6 million Welsh individuals to estimate the prevalence and incidence rates for dystonia and its multiple subtypes. We maintained terminology of Read and ICD‐10 codes for a true reflection of their use within primary and secondary care. Our case ascertainment algorithm demonstrated an overall sensitivity of 79%, identifying 70 of 89 of the clinically confirmed cases. The midyear point prevalence of adult‐onset idiopathic dystonia in Wales increased from 16.4/100,000 (0.02%) in 1994 to 1,219.8/100,000 (1.2%) in 2017, with an increase in annual incidence from 49.9/100,000 (1994) to 96.2/100,000 (2017). Individuals diagnosed with adult‐onset idiopathic dystonia were found to be equally distributed across measures of socioeconomic deprivation at time of diagnosis, with no evidence of change in deprivation over time. There were 4,315 deaths in those diagnosed with adult‐onset idiopathic dystonia, with ischaemic heart disease, cerebrovascular diseases, pneumonia, bronchitis, emphysema, and other chronic obstructive pulmonary diseases being the most common causes of death.

Previous estimates of dystonia prevalence, from service‐ or population‐based studies, have varied between 6.1 and 70.1 per 100,000 in the former [31, 32], to between 7.91 and 732 per 100,000 in the latter [15, 17]. However, these approaches are likely to introduce bias, identifying only those seeking out or receiving clinical care, as well as underascertaining cases, suggesting that rates of dystonia within the population are higher than previously suggested [21]. In support of this, our results suggest an overall prevalence of dystonia syndromes of 1,219.8/100,000 (1.2%), which, given the unbiased nature of the case identification, suggests a more accurate population‐based value. The median age at diagnosis (42 years) and proportion of males to females (1:1.7) determined in this study are consistent with previous studies, suggesting that the cohort identification criteria were appropriately applied.

Amongst population‐based studies, two of the highest notable outliers estimated a prevalence of 732/100,000 (0.73%) and 390/100,000 (0.39%) [6, 17]. Previous UK‐based studies have reported prevalence rates of 38 per 100,000 in those diagnosed with focal dystonia, higher than other European studies reported over the same period [10, 16, 33, 34, 35]. The UK study also used a linkage‐based approach, ascertaining cases by reviewing hospital records, as well as recruiting individuals registered as members to the Dystonia Society, and via a postal survey [36]. Our study has expanded upon this using detailed linked data available across the whole of Wales. More recent epidemiological studies of dystonia show a trend toward elevated estimates compared to the literature prior to 2012 [21], which may be in part due to an ageing population, increased clinical recognition of dystonia, and identification of an ever‐expanding number of causative genes [37].

Three US‐based studies have estimated the incidence of cervical dystonia between 0.8 and 1.18 per 100,000 person‐years [5, 12, 38], and incidence rates of other types of dystonia varied between 0.2 and 0.4/100,000 person‐years [5]. As with the multiple factors influencing prevalence rates, it is likely that these estimates are lower than true population values. In comparison, we found an average annual incidence of 87.65/100,000, increasing from 49.85/100,000 cases (1994) to 96.21/100,000 (2017). New cases were highest between 2004 and 2008, ranging from 104.09 to 112.66 per 100,000. These dates coincide with a known period of higher data capture within SAIL [39], whereas electronic records prior to 2000 in the SAIL Databank are sparse, providing a likely explanation for the more marked increase in numbers after this time. However, rates have slowly decreased since 2011, plateauing at ~100/100,000. Elevated rates of incidence are consistent with increased recognition, awareness, and improved availability of neurological services.

Our study demonstrated that deprivation scores did not change following a diagnosis of dystonia, arguably an unexpected finding given the potential for dystonia to impact employment and social interaction. To our knowledge, there has been no research that directly investigates changes in deprivation, with previous studies that have included measures of this nature identifying conflicting findings. A population‐based survey in India found no significant differences in dystonia prevalence estimates when comparing slum dwellers and non‐slum dwellers, despite their socioeconomic differences [9]. Other studies have also noted that dystonia affects employment, with pain and severity of symptoms impacting work status in up to 40% of cases [40]. However, given that many of the treatments typically used to manage motor symptoms, notably botulinum neurotoxin and deep brain stimulation (DBS), have been shown to improve quality of life, and reduce pain and motor symptom severity, it is plausible that this may contribute to maintained working status [41, 42]. Interestingly, increased rates of DBS surgery have been observed in the USA between 2002 (*n* = 2372) and 2014 (*n* = 5260), with a similar trend likely in the UK [43].

To our knowledge, ours is the first study to assess mortality in patients with dystonia. Of the cohort, 10% died during the study period, with a median age at death of 80 years. There was no evidence of a higher level of premature death (<75 years of age) amongst the dystonia cohort (35.9%), which was comparable to the rate in the general population in Wales (31.7%, 2017). Amongst our cohort, males and females had a median age at death (78 and 82 years, respectively) comparable to the life expectancy in Wales (78.5 years and 82.3 years, 2017–2019) [44]. Males diagnosed with other/unspecified forms of dystonia and writer's cramp were younger than the expected median age (72 and 74 years, respectively), and both males and females (70 and 72.5 years) with idiopathic torsion dystonia were younger at death. The most common causes of death included respiratory disorders, circulatory disorders, cancers, and dementia, in keeping with the leading causes of deaths registered in England and Wales (2017) [45]. Comparable causes of death were also noted amongst dystonia subtypes (Figure 5).

As with all record linkage‐based studies there are several limitations. First, those who have not sought medical attention, either in the community or hospital‐based specialist care, will not be captured by this analysis.

Dystonia is primarily diagnosed in secondary care by a specialist, and subsequently coded in primary care by general practitioners. Our algorithm was 79% sensitive, with 74% of the reference population being identified from primary records alone. Although there is little incentive for GPs to subtype dystonia, in general, there is evidence to suggest that primary care coding is relatively accurate in the UK [46]. Several previous studies, using the SAIL database, have validated the accuracy of using these diagnosis codes for case ascertainment of other neurological conditions [20, 47, 48]. However, coding practices may vary between GPs as well as having the potential to change with time. Our validation cohort consisted of individuals seen in one specialist service, and it may be that these individuals were more likely to get a primary care dystonia code when compared to individuals seen in other services.

There is always the potential for diagnostic misclassification through coding. For example, we did not identify any cases of idiopathic familial dystonia. This likely reflects the focus on achieving the most accurate clinical diagnosis for the individual patient, with information relating to other affected family members not always being known to the patient and/or the treating clinician.

Including hospital inpatient and outpatient data, in addition to primary care data, provided only a small increase in case ascertainment sensitivity (0.9%). This may be because hospital coding is primarily related to recording the admission or attendance, with the reasons for being admitted and comorbidities being less frequently documented [20].

We were unable to obtain a measure of specificity for our case ascertainment algorithm in general and for dystonia subtypes, as we were unable to upload relevant negative control groups. It therefore remains possible that we included a proportion of cases without dystonia. However, we sought to account for this by applying stringent exclusion criteria that would account for most known causes of acquired dystonia or misdiagnosis of dystonia. Lastly, deprivation is difficult to measure, and although the WIMD is one of the more comprehensive deprivation scores available, it does not cover every aspect of deprivation. It is also an area‐based rather than individual‐based measure of deprivation. It is therefore possible that an increase in deprivation for an individual would not change their WIMD score if they did not change address. We may therefore lack recognition of social change in deprivation.

Our results indicate that prevalence and incidence rates of dystonia are higher than previously estimated, 1,220/100,000/year (1.2%) and 96/100,000/year, respectively, potentially indicating more accurate population estimates given the unbiased nature of patient identification. We have shown that a diagnosis of dystonia does not appear to have a detrimental impact on socioeconomic status, with no changes in deprivation observed at follow‐up. In addition, there was no evidence of decreased life expectancy and causes of underlying death, mirroring the Welsh leading causes of death. The findings from this work have important implications for patients, carers, health care providers, third sector organizations, and health care policy, with our case‐ascertainment algorithm providing a platform for application in future population‐based dystonia studies.

## CONFLICT OF INTEREST

The authors report no conflicts of interest.

## AUTHOR CONTRIBUTIONS

**Grace A. Bailey:** Conceptualization (equal), data curation (equal), formal analysis (equal), investigation (equal), methodology (equal), project administration (equal), writing–original draft (equal), writing–review & editing (equal). **Anna Rawlings:** Data curation (lead), formal analysis (supporting), methodology (equal), resources (equal), validation (lead), writing–original draft (equal), writing–review & editing (equal). **Fatemeh Torabi:** Data curation (supporting), formal analysis (equal), resources (equal), writing–review & editing (equal). **Owen Pickrell:** Conceptualization (equal), data curation (equal), formal analysis (supporting), investigation (equal), methodology (equal), project administration (equal), resources (equal), supervision (equal), writing–original draft (equal), writing–review & editing (equal). **Kathryn J. Peall:** Conceptualization (lead), data curation (equal), formal analysis (supporting), funding acquisition (lead), investigation (equal), methodology (equal), project administration (equal), resources (equal), supervision (equal), visualization (equal), writing–original draft (supporting), writing–review & editing (lead).

## ROLE OF THE FUNDER/SPONSOR

The funders had no role in the design or conduct of the study; collection, management, analysis, or interpretation of the data; preparation, review, or approval of the manuscript; or decision to submit the manuscript for publication.

## Data Availability

Data may be obtained from a third party and are not publicly available. The data used in this study are stored within the SAIL Databank at the Health Information Research Unit (HIRU) at Swansea University. All proposals to use SAIL datasets must comply with HIRU’s information governance policy and are subject to review by an independent Information Governance Review Panel (IGRP). Before data can be accessed, approval must be given by the IGRP. SAIL has established an application process to be followed by anyone who would like to access data via SAIL: https://www.saildatabank.com/application‐process.

## References

[1] Junker J , Berman BD , Hall J , et al. Quality of life in isolated dystonia: non‐motor manifestations matter. J Neurol Neurosurg Psychiatry. 2021. 92:622–628. https://jnnp.bmj.com/content/early/2021/02/09/jnnp‐2020‐325193 10.1136/jnnp-2020-325193 PMC8356023 33563813

[2] van den Dool J , Tijssen MAJ , Koelman JHTM , Engelbert RHH , Visser B . Determinants of disability in cervical dystonia. Parkinsonism Relat Disord. 2016;32:48‐53. https://www.sciencedirect.com/science/article/pii/S1353802016303182 27553512 10.1016/j.parkreldis.2016.08.014

[3] Defazio G . The epidemiology of primary dystonia: current evidence and perspectives. Eur J Neurol. 2010;17:9‐14. 10.1111/j.1468-1331.2010.03053.x 20590802

[4] Steeves TD , Day L , Dykeman J , Jette N , Pringsheim T . The prevalence of primary dystonia: a systematic review and meta‐analysis. Mov Disord. 2012;27(14):1789‐1796. 23114997 10.1002/mds.25244

[5] Nutt JG , Muenter MD , Aronson A , Kurland LT , Melton LJ . Epidemiology of focal and generalized dystonia in Rochester, Minnesota. Mov Disord. 1988;3(3):188‐194. 3264051 10.1002/mds.870030302

[6] Jankovic J , Tsui J , Bergeron C . Prevalence of cervical dystonia and spasmodic torticollis in the United States general population. Park Relat Disord. 2007;13(7):411‐416. 10.1016/j.parkreldis.2007.02.005 17442609

[7] Ortiz R , Scheperjans F , Mertsalmi T , Pekkonen E . The prevalence of adult‐onset isolated dystonia in Finland 2007–2016. Fonoff ET, editor. PLoS One. 2018;13(11):e0207729. https://dx.plos.org/ 10.1371/journal.pone.0207729 30458031 PMC6245745

[8] Sugawara M , Watanabe S , Toyoshima I . Prevalence of dystonia in Akita prefecture in northern Japan. Mov Disord. 2006;21(7):1047‐1049. 16602106 10.1002/mds.20880

[9] Das SK , Banerjee TK , Biswas A , et al. Community survey of primary dystonia in the City of Kolkata. India. 2007;22(14):2031‐2036. 10.1002/mds.21446 17722044

[10] Asgeirsson H , Jakobsson F , Hjaltason H , Jonsdottir H , Sveinbjornsdottir S . Prevalence study of primary dystonia in Iceland. Mov Disord. 2006;21(3):293‐298. 16211610 10.1002/mds.20674

[11] Cossu G , Mereu A , Deriu M , et al. Prevalence of primary blepharospasm in Sardinia, Italy: a service‐based survey. Mov Disord. 2006;21(11):2005‐2008. 16960861 10.1002/mds.21084

[12] Marras C , Van Den Eeden SK , Fross RD , et al. Minimum incidence of primary cervical dystonia in a multiethnic health care population. Neurology. 2007;69(7):676‐680. https://pubmed.ncbi.nlm.nih.gov/17698789/ 17698789 10.1212/01.wnl.0000267425.51598.c9

[13] LaHue SC , Albers K , Goldman S , et al. Cervical dystonia incidence and diagnostic delay in a multiethnic population. Mov Disord. 2020;35(3):450‐456. 31774238 10.1002/mds.27927 PMC10683845

[14] Risch N , de Leon D , Ozelius L , et al. Genetic analysis of idiopathic torsion dystonia in Ashkenazi Jews and their recent descent from a small founder population. Nat Genet. 1995;9(2):152‐159. http://www.nature.com/naturegenetics 7719342 10.1038/ng0295-152

[15] Li SC , Schoenberg BS , Wang CC , et al. A prevalence survey of parkinson’s disease and other movement disorders in the people’s Republic of China. Arch Neurol. 1985;42(7):655‐657. https://jamanetwork.com/ 4015461 10.1001/archneur.1985.04060070045013

[16] Castelon Konkiewitz E , Trender‐Gerhard I , Kamm C , et al. Service‐based survey of dystonia in Munich [Internet]. Neuroepidemiology. 2002;21(4):202‐206. www.karger.com 12065883 10.1159/000059525

[17] Müller J , Kiechl S , Wenning GK , et al. The prevalence of primary dystonia in the general community. Neurology. 2002;59(6):941‐943. https://n.neurology.org/content/59/6/941 12297587 10.1212/01.wnl.0000026474.12594.0d

[18] Pickrell WO , Lacey AS , Bodger OG , et al. Epilepsy and deprivation, a data linkage study. Epilepsia. 2015;56(4):585‐591. 25873180 10.1111/epi.12942

[19] Lee SC , DelPozo‐Banos M , Lloyd K , et al. Area deprivation, urbanicity, severe mental illness and social drift — A population‐based linkage study using routinely collected primary and secondary care data. Schizophr Res. 2020;1(220):130‐140. 10.1016/j.schres.2020.03.044 32249120

[20] Carter B , Bennett CV , Bethel J , Jones HM , Wang T , Kemp A . Identifying cerebral palsy from routinely‐collected data in England and Wales. Clin Epidemiol. 2019;11 :457‐468. /pmc/articles/PMC6556471/. 31239784 10.2147/CLEP.S200748 PMC6556471

[21] Defazio G , Berardelli A . Is adult‐onset dystonia a rare disease? Time for population‐based studies. Mov Disord. 2021;36(5):1119‐1124. https://onlinelibrary.wiley.com/doi/ 10.1002/mds.28560 33724530

[22] Office for National Statistics . Population estimates for the UK, England and Wales, Scotland and Northern Ireland ‐ Office for National Statistics [Internet]. Office for National Statistics. 2020. p. 1. https://www.ons.gov.uk/peoplepopulationandcommunity/populationandmigration/populationestimates/bulletins/annualmidyearpopulationestimates/mid2019estimates%0Ahttps://www.ons.gov.uk/peoplepopulationandcommunity/populationandmigration/populationestimates/bul. Accessed January 20, 2021.

[23] Wolf A , Dedman D , Campbell J , et al. Data resource profile: clinical practice research datalink (CPRD) Aurum. Int J Epidemiol. 2019;48(6):1740‐1740G. https://academic.oup.com/ije/article/48/6/1740/5374844 30859197 10.1093/ije/dyz034 PMC6929522

[24] Correll P , Feyer A‐M , Phan P‐T , et al. Lumos: a statewide linkage programme in Australia integrating general practice data to guide system redesign. Integ Heal J. 2021;3:74. http://ihj.bmj.com/ 10.1136/ihj-2021-000074 PMC10240716 37441059

[25] Jones KH , Ford DV , Thompson S , Lyons R . A profile of the SAIL databank on the UK secure research platform. Int J Popul Data Sci. 2019;4(2):1134. 34095541 10.23889/ijpds.v4i2.1134 PMC8142954

[26] Lyons RA , Jones KH , John G , et al. The SAIL databank: linking multiple health and social care datasets. BMC Med Inform Decis Mak. 2009;9(1):1472‐6947. http://www.biomedcentral.com/1472‐6947/9/3 10.1186/1472-6947-9-3 PMC2648953 19149883

[27] Ichinose H , Ohye T , Takahashi E , et al. Hereditary progressive dystonia with marked diurnal fluctuation caused by mutations in the GTP cyclohydrolase I gene. Nat Genet. 1994;8(3):236‐242. http://www.nature.com/naturegenetics 7874165 10.1038/ng1194-236

[28] Welsh Government . Welsh Index of Multiple Deprivation (WIMD) 2014 ‐ Summary: Revised [Internet]. 2014. Available from: http://www.nationalarchives.gov.uk/doc/open‐government‐licence/version/3/. Accessed Mar 23, 2021.

[29] Hellberg C , Alinder E , Jaraj D , Puschmann A . Nationwide prevalence of primary dystonia, progressive ataxia and hereditary spastic paraplegia. Park Relat Disord. 2019;1(69):79‐84. 10.1016/j.parkreldis.2019.10.028 31706130

[30] Martino D , Brander G , Svenningsson P , Larsson H , de la Cruz LF . Association and familial coaggregation of idiopathic dystonia with psychiatric outcomes. Mov Disord. 2020;35(12):2270‐2278. https://onlinelibrary.wiley.com 32940390 10.1002/mds.28257

[31] Nakashima K , Kusumi M , Inoue Y , Takahashi K . Prevalence of focal dystonias in the western area of Tottori Prefecture in Japan. Mov Disord. 1995;10(4):440‐443. 7565823 10.1002/mds.870100406

[32] Atehortúa JMS , Jaramillo SPI , Bañol AR , Henao OB . Prevalence of dystonia in Antioquia, Colombia. Neuroepidemiology. 2016;46(2):137‐143. www.karger.com/ned 26820666 10.1159/000443834

[33] Butler AG , Duffey PO , Hawthorne MR , Barnes MP . An epidemiologic survey of dystonia within the entire population of northeast England over the past nine years. Adv Neurol. 2004;94:95‐99. https://pubmed.ncbi.nlm.nih.gov/14509660/ 14509660

[34] Defazio G , Livrea P , De Salvia R , et al. Prevalence of primary blepharospasm in a community of Puglia region, Southern Italy. Neurology. 2001;56(11):1579‐1581. 11402121 10.1212/wnl.56.11.1579

[35] Le KD , Nilsen B , Dietrichs E . Prevalence of primary focal and segmental dystonia in Oslo. Neurology. 2003;61(9):1294‐1296. 14610144 10.1212/01.wnl.0000090463.05980.59

[36] Duffey PO , Butler AG , Hawthorne MR , Barnes MP . The epidemiology of the primary dystonias in the north of England. Adv Neurol. 1998;78:121‐125. https://pubmed.ncbi.nlm.nih.gov/9750909/ 9750909

[37] Lohmann K , Klein C . Update on the genetics of dystonia. Curr Neurol Neurosci Rep. 2017;17(3):26. www.omim.org 28283962 10.1007/s11910-017-0735-0

[38] LaHue SC , Albers K , Goldman S , et al. Cervical dystonia incidence and diagnostic delay in a multiethnic population. Mov Disord. 2020;35(3):450‐456. https://onlinelibrary.wiley.com/doi/abs/ 31774238 10.1002/mds.27927 PMC10683845

[39] Demmler JC , Brophy ST , Marchant A , John A , Tan JOA . Shining the light on eating disorders, incidence, prognosis and profiling of patients in primary and secondary care: national data linkage study. Br J Psychiatry. 2020;216(2):105‐112. 31256764 10.1192/bjp.2019.153 PMC7557634

[40] Molho ES , Stacy M , Gillard P , et al. Impact of cervical dystonia on work productivity: an analysis from a patient registry. Mov Disord Clin Pract. 2016;3(2):130‐138. www.interscience.wiley.com 27774495 10.1002/mdc3.12238 PMC5064605

[41] Moll M , Rosenthal D , Hefter H . Quality of life in long‐term botulinum toxin treatment of cervical dystonia: results of a cross sectional study. Park Relat Disord. 2018;1(57):63‐67. 10.1016/j.parkreldis.2018.07.019 30150129

[42] Tsuboi T , Wong JK , Okun MS , Ramirez‐Zamora A . Quality of life outcomes after deep brain stimulation in dystonia: a systematic review. Parkinsonism Relat Disord. Elsevier Ltd. 2020;70:82‐93. 31767450 10.1016/j.parkreldis.2019.11.016 PMC9080959

[43] Deng H , Yue JK , Wang DD . Trends in safety and cost of deep brain stimulation for treatment of movement disorders in the United States: 2002–2014. Br J Neurosurg. 2021;35(1):57‐64. https://www.tandfonline.com/action/journalInformation?journalCode=ibjn20 32476485 10.1080/02688697.2020.1759776

[44] Office for National Statistics . Life expectancy by National Assembly Wales constituency and National Assembly Wales electoral region, 2017 to 2019 ‐ Office for National Statistics [Internet]. Office for National Statistics. 2021. https://www.ons.gov.uk/peoplepopulationandcommunity/birthsdeathsandmarriages/lifeexpectancies/adhocs/12983lifeexpectancybynationalassemblywalesconstituencyandnationalassemblywaleselectoralregion2017to2019. Accessed April 13, 2021.

[45] Office for National Statistics . Deaths registered in England and Wales (series DR) ‐ Office for National Statistics [Internet]. Office for National Statistics. 2017. https://www.ons.gov.uk/peoplepopulationandcommunity/birthsdeathsandmarriages/deaths/bulletins/deathsregisteredinenglandandwalesseriesdr/2017#suicide‐accounted‐for‐an‐increased‐proportion‐of‐deaths‐at‐ages‐5‐to‐19‐years‐in‐2017%0Ahttps://www.ons.gov.uk/peo. Accessed April 13, 2021.

[46] Herrett E , Thomas SL , Marieke Schoonen W , Smeeth L , Hall AJ , Emily HM . Validation and validity of diagnoses in the general practice research database: a systematic review. Br J Clin Pharmacol. 2010;69(1):4‐14. http://www3.interscience.wiley.com/ 20078607 10.1111/j.1365-2125.2009.03537.x PMC2805870

[47] Fonferko‐Shadrach B , Lacey AS , White CP , et al. Validating epilepsy diagnoses in routinely collected data. Seizure. 2017;52:195‐198. /pmc/articles/PMC5703030/. 29059611 10.1016/j.seizure.2017.10.008 PMC5703030

[48] Miah L , Strafford H , Fonferko‐Shadrach B , et al. Incidence, prevalence and healthcare outcomes in idiopathic intracranial hypertension: a population study. Neurology. 2021;96(8):e1251‐e1261. doi:10.1212/WNL.0000000000011463 PMC8055349 33472926

