# Supplementary material for: Adult‐onset idiopathic dystonia: A national data‐linkage study to determine epidemiological, social deprivation, and mortality characteristics
Source: Eur J Neurol. 2021 Oct 15;29(1):91–104. doi: 10.1111/ene.15114 (PMC9377012; doi:10.1111/ene.15114)
Supplement: Supplementary file 1 [file ENE-29-91-s001.docx]

**Supplementary Table 1. Different combinations of Read codes to develop case ascertainment algorithm using Welsh Longitudinal General Practice dataset**

| **Algorithm 1** | | | | **Algorithm 2** | | | | **Algorithm 3** | | | | **Algorithm 4** | | | | **Algorithm 5** | | | |
| --- | --- | --- | --- | --- | --- | --- | --- | --- | --- | --- | --- | --- | --- | --- | --- | --- | --- | --- | --- |
| **Read Code** | **Description** | **Level** | **Sensitivity (n)** | **Read Code** | **Description** | **Level** | **Sensitivity (n)** | **Read Code** | **Description** | **Level** | **Sensitivity (n)** | **Read Code** | **Description** | **Level** | **Sensitivity (n)** | **Read Code** | **Description** | **Level** | **Sensitivity (n)** |
| 16A3. | Torticollis - symptom | 1 | 73.7% (59) | 16A3. | Torticollis - symptom | 1 | 73.7% (59) | 16A3. | Torticollis - symptom | 1 | 73.7% (59) | 16A3. | Torticollis - symptom | 1 | 73.7% (59) | 16A3. | Torticollis - symptom | 1 | 73.7% (59) |
| F136. | Idiopathic torsion dystonia |  |  | F136. | Idiopathic torsion dystonia |  |  | F136. | Idiopathic torsion dystonia |  |  | F136. | Idiopathic torsion dystonia |  |  | F136. | Idiopathic torsion dystonia |  |  |
| F1360 | Idiopathic familial dystonia |  |  | F1360 | Idiopathic familial dystonia |  |  | F1360 | Idiopathic familial dystonia |  |  | F1360 | Idiopathic familial dystonia |  |  | F1360 | Idiopathic familial dystonia |  |  |
| F137. | Symptomatic torsion dystonia |  |  | F137. | Symptomatic torsion dystonia |  |  | F137. | Symptomatic torsion dystonia |  |  | F137. | Symptomatic torsion dystonia |  |  | F137. | Symptomatic torsion dystonia |  |  |
| F1380 | Blepharospasm |  |  | F1380 | Blepharospasm |  |  | F1380 | Blepharospasm |  |  | F1380 | Blepharospasm |  |  | F1380 | Blepharospasm |  |  |
| F1382 | Spasmodic torticollis |  |  | F1382 | Spasmodic torticollis |  |  | F1382 | Spasmodic torticollis |  |  | F1382 | Spasmodic torticollis |  |  | F1382 | Spasmodic torticollis |  |  |
| F1383 | Organic writers' cramp |  |  | F1383 | Organic writers' cramp |  |  | F1383 | Organic writers' cramp |  |  | F1383 | Organic writers' cramp |  |  | F1383 | Organic writers' cramp |  |  |
| F138z | Torsion dystonia fragm. NOS |  |  | F138z | Torsion dystonia fragm. NOS |  |  | F138z | Torsion dystonia fragm. NOS |  |  | F138z | Torsion dystonia fragm. NOS |  |  | F138z | Torsion dystonia fragm. NOS |  |  |
| F13A. | Paroxysmal dystonia |  |  | F13A. | Paroxysmal dystonia |  |  | F13A. | Paroxysmal dystonia |  |  | F13A. | Paroxysmal dystonia |  |  | F13B. | Myoclonic dystonia |  |  |
| F13B. | Myoclonic dystonia |  |  | F13B. | Myoclonic dystonia |  |  | F13B. | Myoclonic dystonia |  |  | F13B. | Myoclonic dystonia |  |  | F13C. | Segawa syndrome |  |  |
| F13C. | Segawa syndrome |  |  | F13C. | Segawa syndrome |  |  | F13C. | Segawa syndrome |  |  | F13C. | Segawa syndrome |  |  | F13X. | Dystonia, unspecified |  |  |
| F13X. | Dystonia, unspecified |  |  | F13X. | Dystonia, unspecified |  |  | F13X. | Dystonia, unspecified |  |  | F13X. | Dystonia, unspecified |  |  | Fyu24 | [X]Other dystonia |  |  |
| Fyu24 | [X]Other dystonia |  |  | Fyu24 | [X]Other dystonia |  |  | Fyu24 | [X]Other dystonia |  |  | Fyu24 | [X]Other dystonia |  |  | Fyu2A | [X]Dystonia, unspecified |  |  |
| Fyu2A | [X]Dystonia, unspecified |  |  | Fyu2A | [X]Dystonia, unspecified |  |  | Fyu2A | [X]Dystonia, unspecified |  |  | Fyu2A | [X]Dystonia, unspecified |  |  | N135. | Torticollis unspecified |  |  |
| N135. | Torticollis unspecified |  |  | N135. | Torticollis unspecified |  |  | N135. | Torticollis unspecified |  |  | N135. | Torticollis unspecified |  |  | N1350 | Intermittent torticollis |  |  |
| N1350 | Intermittent torticollis |  |  | N1350 | Intermittent torticollis |  |  | N1350 | Intermittent torticollis |  |  | N1350 | Intermittent torticollis |  |  | N135z | Torticollis NOS |  |  |
| N135z | Torticollis NOS |  |  | N135z | Torticollis NOS |  |  | N135z | Torticollis NOS |  |  | N135z | Torticollis NOS |  |  | F137y | Symptomat torsion dystonia OS | 2 | 82.5 (66) |
| F131. | Essent.+other specified tremor | 2: medication and diagnosis  2: diagnosis | 83.75 (67)  83.75 (67) | F131. | Essent.+other specified tremor | 2: diagnosis  2: diagnosis and medication | 82.5% (66)  82.5% (66) | F131. | Essent.+other specified tremor | 2: diagnosis  2: diagnosis and medication | 82.5% (66)  82.5% (66) | F131. | Essent.+other specified tremor | 2 |  | F137z | Symptomat torsion dystonia NOS |  |  |
| F1310 | Benign essential tremor |  |  | F1310 | Benign essential tremor |  |  | F1310 | Benign essential tremor |  |  | F1310 | Benign essential tremor |  |  | F138. | Fragments of torsion dystonia |  |  |
| F1372 | Drug-induced dystonia |  |  | F137y | Symptomat torsion dystonia OS |  |  | F137y | Symptomat torsion dystonia OS |  |  | F137y | Symptomat torsion dystonia OS |  |  | 1B22. | Has a tremor |  |  |
| F137y | Symptomat torsion dystonia OS |  |  | F137z | Symptomat torsion dystonia NOS |  |  | F137z | Symptomat torsion dystonia NOS |  |  | F137z | Symptomat torsion dystonia NOS |  |  |  |  |  |  |
| F137z | Symptomat torsion dystonia NOS |  |  | F138. | Fragments of torsion dystonia |  |  | F138. | Fragments of torsion dystonia |  |  | F138. | Fragments of torsion dystonia |  |  |  |  |  |  |
| F138. | Fragments of torsion dystonia |  |  | 1B22. | Has a tremor |  |  | 1B22. | Has a tremor |  |  | 1B22. | Has a tremor |  |  |  |  |  |  |
| 1B22. | Has a tremor |  |  | F131z | Essential and other specified forms of tremor |  |  | F131z | Essential and other specified forms of tremor |  |  | F131z | Essential and other specified forms of tremor |  |  |  |  |  |  |
| 1B25. | Has spasms |  |  | ds3.. | CLOSTRIDIUM BOTULINUM A T-HC |  |  | ds3.. | CLOSTRIDIUM BOTULINUM A T-HC |  |  |  |  |  |  |  |  |  |  |
| F131z | Essential and other specified forms of tremor |  |  | ds31. | *BOTULINUM A D-HC 500units inj |  |  | ds31. | *BOTULINUM A D-HC 500units inj |  |  |  |  |  |  |  |  |  |  |
| ds3.. | CLOSTRIDIUM BOTULINUM A T-HC |  |  | ds32. | DYSPORT 500units injection |  |  | ds32. | DYSPORT 500units injection |  |  |  |  |  |  |  |  |  |  |
| ds31. | *BOTULINUM A D-HC 500units inj |  |  | ds33. | BOTOX 100units inj powder |  |  | ds33. | BOTOX 100units inj powder |  |  |  |  |  |  |  |  |  |  |
| ds32. | DYSPORT 500units injection |  |  | ds34. | *CLOSTRIDIUM BOTUL 100u inj |  |  | ds34. | *CLOSTRIDIUM BOTUL 100u inj |  |  |  |  |  |  |  |  |  |  |
| ds33. | BOTOX 100units inj powder |  |  | ds36. | *CLOSTRIDIUM BOTUL 50u inj pdr |  |  | ds36. | *CLOSTRIDIUM BOTUL 50u inj pdr |  |  |  |  |  |  |  |  |  |  |
| ds34. | *CLOSTRIDIUM BOTUL 100u inj |  |  | ds37. | XEOMIN 100units pdr for inj |  |  | ds37. | XEOMIN 100units pdr for inj |  |  |  |  |  |  |  |  |  |  |
| ds36. | *CLOSTRIDIUM BOTUL 50u inj pdr |  |  | ds38. | BOTOX 50units pdr for inj |  |  | ds38. | BOTOX 50units pdr for inj |  |  |  |  |  |  |  |  |  |  |
| ds37. | XEOMIN 100units pdr for inj |  |  | ds3C. | XEOMIN 50units pdr for inj |  |  | ds3C. | XEOMIN 50units pdr for inj |  |  |  |  |  |  |  |  |  |  |
| ds38. | BOTOX 50units pdr for inj |  |  | T8531 | Accid.pois.-anticholinesterase |  |  | 7Q040 | Torsion dystonias other involuntary movements drugs Band 1 |  |  |  |  |  |  |  |  |  |  |
| ds3B. | DYSPORT 300units injection |  |  | T854. | Accid.pois.- anticholinergics |  |  |  |  |  |  |  |  |  |  |  |  |  |  |
| ds3C. | XEOMIN 50units pdr for inj |  |  | T854z | Accid.pois.- anticholinerg.NOS |  |  |  |  |  |  |  |  |  |  |  |  |  |  |
| T8531 | Accid.pois.-anticholinesterase |  |  | TJ953 | AR - tetrabenazine |  |  |  |  |  |  |  |  |  |  |  |  |  |  |
| T854. | Accid.pois.- anticholinergics |  |  | TJB05 | AR - anticholinesterase |  |  |  |  |  |  |  |  |  |  |  |  |  |  |
| T854z | Accid.pois.- anticholinerg.NOS |  |  | 7Q040 | Torsion dystonias other involuntary movements drugs Band 1 |  |  |  |  |  |  |  |  |  |  |  |  |  |  |
| TJ953 | AR - tetrabenazine |  |  | F1311 | Familial tremor | 3: diagnosis  3: diagnosis and medication | 83.75% (67)  87.5% (70) |  |  |  |  |  |  |  |  |  |  |  |  |
| TJB05 | AR - Anticholinesterase |  |  | H1y74 | Laryngeal spasm |  |  |  |  |  |  |  |  |  |  |  |  |  |  |
| 7Q040 | Torsion dystonias other involuntary movements drugs Band 1 |  |  | R0102 | [D]Spasms NOS |  |  |  |  |  |  |  |  |  |  |  |  |  |  |
| F1311 | Familial tremor | 3: diagnosis  3: diagnosis and medication | 85% (68)  88.75% (71) | dn41. | RIVOTRIL 500micrograms tablets |  |  |  |  |  |  |  |  |  |  |  |  |  |  |
| H1y74 | Laryngeal spasm |  |  | dn42. | RIVOTRIL 2mg tablets |  |  |  |  |  |  |  |  |  |  |  |  |  |  |
| F132. | Myoclonus |  |  | dn4w. | CLONAZEPAM 0.5mg/5mL s/f soln |  |  |  |  |  |  |  |  |  |  |  |  |  |  |
| F132y | Myoclonus OS |  |  | dn4x. | CLONAZEPAM 2mg/5mL s/f soln |  |  |  |  |  |  |  |  |  |  |  |  |  |  |
| F132z | Myoclonus NOS |  |  | dn4y. | CLONAZEPAM 500mcg tablets |  |  |  |  |  |  |  |  |  |  |  |  |  |  |
| Fyu27 | [X]O spcf extrpyrmdl+mvmnt dis |  |  | dn4z. | CLONAZEPAM 2mg tablets |  |  |  |  |  |  |  |  |  |  |  |  |  |  |
| Fyu28 | [X]Extrpyrmdl+mvmt diso/dis CE |  |  | j82.. | BACLOFEN |  |  |  |  |  |  |  |  |  |  |  |  |  |  |
| PE1.. | Congenital sternomastoid torticollis |  |  | j821. | LIORESAL 10mg tablets |  |  |  |  |  |  |  |  |  |  |  |  |  |  |
| R0102 | [D]Spasms NOS |  |  | j822. | LIORESAL 5mg/5mL sf liquid |  |  |  |  |  |  |  |  |  |  |  |  |  |  |
| dn41. | RIVOTRIL 500micrograms tablets |  |  | j823. | *BACLOSPAS 10mg tablets |  |  |  |  |  |  |  |  |  |  |  |  |  |  |
| dn42. | RIVOTRIL 2mg tablets |  |  | j827. | LYFLEX 5mg/5mL s/f oral soln |  |  |  |  |  |  |  |  |  |  |  |  |  |  |
| dn4w. | CLONAZEPAM 0.5mg/5mL s/f soln |  |  | j82t. | BACLOFEN 40mg/20mL soln inj |  |  |  |  |  |  |  |  |  |  |  |  |  |  |
| dn4x. | CLONAZEPAM 2mg/5mL s/f soln |  |  | j82v. | BACLOFEN 50mcg/1mL i-t inj |  |  |  |  |  |  |  |  |  |  |  |  |  |  |
| dn4y. | CLONAZEPAM 500mcg tablets |  |  | j82w. | BACLOFEN 10mg/5mL i-t inj |  |  |  |  |  |  |  |  |  |  |  |  |  |  |
| dn4z. | CLONAZEPAM 2mg tablets |  |  | j82w. | BACLOFEN 10mg/5mL i-t inj |  |  |  |  |  |  |  |  |  |  |  |  |  |  |
| j82.. | BACLOFEN |  |  | j82x. | BACLOFEN 10mg/20mL i-t inj |  |  |  |  |  |  |  |  |  |  |  |  |  |  |
| j821. | LIORESAL 10mg tablets |  |  | j82y. | BACLOFEN 10mg tablets |  |  |  |  |  |  |  |  |  |  |  |  |  |  |
| j822. | LIORESAL 5mg/5mL sf liquid |  |  | j82z. | BACLOFEN 5mg/5mL sf liquid |  |  |  |  |  |  |  |  |  |  |  |  |  |  |
| j823. | *BACLOSPAS 10mg tablets |  |  | T8502 | Accid.pois.- levodopa (L-dopa) |  |  |  |  |  |  |  |  |  |  |  |  |  |  |
| j827. | LYFLEX 5mg/5mL s/f oral soln |  |  | TJ641 | AR - levodopa - L-dopa |  |  |  |  |  |  |  |  |  |  |  |  |  |  |
| j82t. | BACLOFEN 40mg/20mL soln inj |  |  |  |  |  |  |  |  |  |  |  |  |  |  |  |  |  |  |
| j82v. | BACLOFEN 50mcg/1mL i-t inj |  |  |  |  |  |  |  |  |  |  |  |  |  |  |  |  |  |  |
| j82w. | BACLOFEN 10mg/5mL i-t inj |  |  |  |  |  |  |  |  |  |  |  |  |  |  |  |  |  |  |
| j82x. | BACLOFEN 10mg/20mL i-t inj |  |  |  |  |  |  |  |  |  |  |  |  |  |  |  |  |  |  |
| j82y. | BACLOFEN 10mg tablets |  |  |  |  |  |  |  |  |  |  |  |  |  |  |  |  |  |  |
| j82z. | BACLOFEN 5mg/5mL sf liquid |  |  |  |  |  |  |  |  |  |  |  |  |  |  |  |  |  |  |
| T8502 | Accid.pois.- levodopa (L-dopa) |  |  |  |  |  |  |  |  |  |  |  |  |  |  |  |  |  |  |
| TJ641 | AR - levodopa - L-dopa |  |  |  |  |  |  |  |  |  |  |  |  |  |  |  |  |  |  |
| E2601 | Psychogenic torticollis | 4 | 88.75% (71) |  |  |  |  |  |  |  |  |  |  |  |  |  |  |  |  |
| Eu45y | Psychogenic torticollis - other somatoform disorders |  |  |  |  |  |  |  |  |  |  |  |  |  |  |  |  |  |  |
| F312. | Clonic hemifacial spasm |  |  |  |  |  |  |  |  |  |  |  |  |  |  |  |  |  |  |
| N23y4 | Spasm of muscle |  |  |  |  |  |  |  |  |  |  |  |  |  |  |  |  |  |  |
| N23yE | Spasm of back muscles |  |  |  |  |  |  |  |  |  |  |  |  |  |  |  |  |  |  |
