# Supplementary material for: Adult‐onset idiopathic dystonia: A national data‐linkage study to determine epidemiological, social deprivation, and mortality characteristics
Source: Eur J Neurol. 2021 Oct 15;29(1):91–104. doi: 10.1111/ene.15114 (PMC9377012; doi:10.1111/ene.15114)
Supplement: Supplementary file 2 [file ENE-29-91-s003.docx]

**Supplementary Table 2. Read Codes and ICD-10 codes used to exclude any potential secondary causes of dystonia**

| **Clinical Terminology** | **Read code** | **ICD-10 code** |
| --- | --- | --- |
| *Dystonia* |  |  |
| Paroxysmal dystonia | F13A. |  |
| Drug induced dystonia | F1312 | G240 |
| *Parkinson’s Disease and secondary parkinsonism* | |  |
| Parkinson's Disease | F12.. | G20 |
| Parkinson's disease NOS | F12z. |  |
| O/E Parkinson gait | 2994. |  |
| O/E - Parkinson posture | 2987. |  |
| O/E - Parkinsonian tremor | 297A. |  |
| Dementia in Parkinson's disease | Eu023 |  |
| FH: Parkinsonism | 129Z. |  |
| Secondary parkinsonism due to other external agents | F12W. |  |
| Parkinsonism secondary to drugs | F121. |  |
| Malignant neuroleptic syndrome | F122. |  |
| Postencephalitic parkinsonism | F123. |  |
| Vascular parkinsonism | F124. |  |
| Syphilitic parkinsonism | A94y1 |  |
| Secondary parkinsonism, unspecified | F12X. |  |
| Secondary parkinsonism |  | G21 |
| History of Parkinson's disease | 147F. |  |
| Cerebral degeneration in Parkinson's disease | F11x9 |  |
| *Huntington’s Disease* | | G10 |
| Huntington's chorea | F134. |  |
| Dementia in Huntington's disease | Eu022 |  |
| FH: Huntington’s chorea | 1291. |  |
| *Chorea* | |  |
| Other choreas | F135. |  |
| Hemiballismus | F1350 |  |
| Paroxysmal chorea-athetosis | F1351 |  |
| Drug-induced chorea | F1352 |  |
| Other choreas NOS | F135z |  |
| *Ataxia* | |  |
| Cerebral ataxia | F11y1 |  |
| Cerebellar ataxia NOS | F143. |  |
| Cerebellar ataxia in diseases EC | F144. |  |
| Cerebellar ataxia due to alcoholism | F1440 |  |
| Cerebellar ataxia due to myxoedema | F1441 |  |
| Cerebellar ataxia due to neoplasia | F1442 |  |
| Cerebellar ataxia in disease NOS | F144z |  |
| Congenital nonprogressive ataxia | F145. |  |
| Early onset cerebellar ataxia with hypogonadism | F146. |  |
| Friedreich's ataxia | F140. |  |
| Spinocerebellar disease | F14.. |  |
| Spinocerebellar disease NOS | F14z. |  |
| Other spinocerebellar diseases | F14y. |  |
| Hereditary ataxia |  | G11 |
| *Degenerative diseases of the basal ganglia* | | G23 |
| Other basal ganglia degenerative diseases | F130. |  |
| Dejerine-Thomas syndrome | F1300 |  |
| Hallervorden-Spatz disease | F1301 |  |
| Striatonigral degeneration | F1302 |  |
| Parkinsonism with orthostatic hypotension | F1303 |  |
| Progressive supranuclear ophthalmoplegia | F1304 |  |
| Shy-Drager syndrome | F1305 |  |
| Aicardi Goutieres syndrome | F1306 |  |
| Other basal ganliga degenerative diseases NOS | F130z |  |
| Steele-Richardson-Olszewski syndrome | F24y2 |  |
| *Myoclonus* | |  |
| Myoclonus | F132. |  |
| Familial essential myoclonus | F1320 |  |
| Progressive myoclonic epilepsy | F1321 |  |
| Myoclonic encephalopathy | F1322 |  |
| Myoclonic jerks | F1323 |  |
| Other specified myoclonus | F132y |  |
| Myoclonus NOS | F132z |  |
| O/E myoclonus | 2979. |  |
| Benign neonatal sleep myoclonus | F13z5 |  |
| *Extrapyramidal diseases and movement disorders* |  |  |
| Other extrapyramidal diseases of basal ganglia |  | G25 |
| Extrapyramidal and movement disorders in diseases classified elsewhere |  | G26 |
| Other extrapyramidal disease and abnormal movement disorders | F13.. |  |
| Other/unspecified extrapyramidal/abnormal movement disorders | F13z. |  |
| Unspecified extrapyramidal disease | F13z0 |  |
| Stiff-man syndrome | F13z1 |  |
| Restless leg syndrome | F13z2 |  |
| Akinetic rigid syndrome | F13z3 |  |
| Hyperekplexia | F13z4 |  |
| Neuroferritinopathy | F13z6 |  |
| Extrapyramidal disease and abnormal movement disorder NOS | F13zz |  |
| Other/unspecified extrapyramidal/abnormal movement disorders | F139. |  |
| Paroxysmal non-kinesigenic dyskinesia | F1390 |  |
| Paroxysmal kinesigenic dyskinesia | F1391 |  |
| *Essential and other specified forms of tremor* | F131. |  |
| Benign essential tremor | F1310 |  |
| Familial tremor | F1311 |  |
| Drug-induced tremor | F1312 |  |
| Essential and other specified forms of tremor NOS | F131z |  |
| *Degenerative diseases of the nervous system* |  |  |
| Other degenerative diseases of nervous system, not elsewhere classified |  | G31 |
| *Cerebral degenerations usually manifest in childhood* | F10.. |  |
| Leucodystrophy | F100. |  |
| Krabbe's disease | F1000 |  |
| Schulz's disease | F1001 |  |
| Pelizaeus-Merzbacher disease | F1002 |  |
| Leucodystrophy NOS | F100z |  |
| Cerebral lipidoses | F101. |  |
| Jansky-Bielschowsky disease | F1010 |  |
| Kuf's disease | F1011 |  |
| Spielmeyer-Vogt (Batten) disease | F1012 |  |
| Tay-Sach’s disease | F1013 |  |
| Gangliosidosis | F1014 |  |
| Retinal dystrophy in cerebroretinal lipidosis | F1015 |  |
| Sandhoff disease | F1016 |  |
| Cerebral lipidoses NOS | F101z |  |
| Cerebral degeneration in lipidoses EC | F102. |  |
| Cerebral degeneration in Gaucher's disease | F1020 |  |
| Cerebral degeneration in Niemann-Pick disease | F1021 |  |
| Cerebral degeneration in lipidosis NOS | F102z |  |
| Cerebral degeneration in diseases EC | F103. |  |
| Cerebral degeneration in Hunter's disease | F1030 |  |
| Cerebral degeneration in mucopolysaccharidoses | F1031 |  |
| Cerebral degeneration in disease NOS | F103z |  |
| Other cerebral degenerations in childhood | F10y. |  |
| Alper's disease | F10y0 |  |
| Leigh's disease | F10y1 |  |
| PEHO syndrome | F10y2 |  |
| Other cerebral degenerations in childhood NOS | F10yz |  |
| Childhood cerebral degenerations NOS | F10z. |  |
| *Other cerebral degenerations* | F11.. |  |
| Alzheimer's disease | F110. |  |
| Alzheimer's disease with early onset | F1100 |  |
| Alzheimer's disease with late onset | F1101 |  |
| Pick’s disease | F111. |  |
| Senile degeneration of brain | F112. |  |
| Acquired communicating hydrocephalus | F113. |  |
| Normal pressure hydrocephalus | F1130 |  |
| Communicating hydrocephalus - acquired NOS | F113z |  |
| Acquired obstructive hydrocephalus | F114. |  |
| Hydrocephalus | F115. |  |
| Lewy body disease | F116. |  |
| Infantile posthaemorrhagic hydrocephalus | F117. |  |
| Frontotemporal degeneration | F118. |  |
| Post-traumatic hydrocephalus, unspecified | F11X. |  |
| Cerebral degeneration in other disease EC | F11x. |  |
| Cerebral degeneration due to alcoholism | F11x0 |  |
| Cerebral degeneration due to beriberi | F11x1 |  |
| Cerebral degeneration due to cerebrovascular disease | F11x2 |  |
| Cerebral degeneration due to congenital hydrocephalus | F11x3 |  |
| Cerebral degeneration due to neoplastic disease | F11x4 |  |
| Cerebral degeneration due to myxoedema | F11x5 |  |
| Cerebral degeneration due to vitamin B12 deficiency | F11x6 |  |
| Cerebral degeneration due to Jakob - Creutzfeldt disease | F11x7 |  |
| Cerebral degeneration due to progressive multifocal leukoencephalopathy | F11x8 |  |
| Cerebral degeneration other disease NOS | F11xz |  |
| Other cerebral degeneration | F11y. |  |
| Reye's syndrome | F11y0 |  |
| Corticobasal degeneration | F11y2 |  |
| Other cerebral degeneration NOS | F11yz |  |
| Cerebral degeneration NOS | F11z. |  |
| Hereditary spastic paraplegia | F141. |  |
| Primary cerebellar degeneration | F142. |  |
| Marie's cerebellar ataxia | F1420 |  |
| Sanger-Brown cerebellar ataxia | F1421 |  |
| Dyssynergia cerebellaris myoclonica | F1422 |  |
| Primary cerebellar degeneration NOS | F142z |  |
| *Other spinocerebellar diseases* | F14y. |  |
| Ataxia-telangiectasia | F14y0 |  |
| Corticostriatal-spinal degeneration | F14y1 |  |
| Other spinocerebellar disease NOS | F14yz |  |
| *Anterior horn cell disease* | F15.. |  |
| Werdnig - Hoffmann disease | F150. |  |
| Spinal muscular atrophy | F151. |  |
| Unspecified spinal muscular atrophy | F1510 |  |
| Kugelberg - Welander disease | F1511 |  |
| Adult spinal muscular atrophy | F1512 |  |
| X-linked bulbo-spinal atrophy | F1513 |  |
| Spinal muscular atrophy NOS | F151z |  |
| Other anterior horn cell disease | F15y. |  |
| Anterior horn cell disease NOS | F15z. |  |
| Motor neurone disease | F152. |  |
| Amyotrophic lateral sclerosis | F1520 |  |
| Progressive muscular atrophy | F1521 |  |
| Progressive bulbar palsy | F1522 |  |
| Pseudobulbar palsy | F1523 |  |
| Primary lateral sclerosis | F1524 |  |
| Motor neurone disease NOS | F152z |  |
| *Other diseases of spinal cord* | F16.. |  |
| Syringomyelia and syringobulbia | F160. |  |
| Syringomyelia | F1600 |  |
| Syringobulbia | F1601 |  |
| Syringomyelia or syringobulbia NOS | F160z |  |
| Vascular myelopathies | F161. |  |
| Myelopathy due to acute infarction of spinal cord | F1610 |  |
| Myelopathy due to arterial thrombosis of spinal cord | F1611 |  |
| Myelopathy due to oedema of spinal cord | F1612 |  |
| Myelopathy due to haematomyelia | F1613 |  |
| Subacute necrotic myelopathy | F1614 |  |
| Anterior spinal artery thrombosis | F1615 |  |
| Vascular myelopathy NOS | F161z |  |
| Subacute combined degeneration of spinal cord | F162. |  |
| Myelopathy due to disease EC | F163. |  |
| Myelopathy due to intervertebral disc disease | F1630 |  |
| Myelopathy due to neoplastic disease | F1631 |  |
| Myelopathy due to spondylosis | F1632 |  |
| Myelopathy due to disease NOS | F163z |  |
| Brown-Sequard syndrome | F164. |  |
| Other myelopathy | F16y. |  |
| Drug induced myelopathy | F16y0 |  |
| Radiation induced myelopathy | F16y1 |  |
| Other myelopathy NOS | F16yz |  |
| Myelopathy NOS | F16z. |  |
| *Hereditary and degenerative diseases of the CNS OS* | F1y.. |  |
| Fragile X associated tremor ataxia syndrome | F1y0. |  |
| Hereditary and degenerative diseases of the central nervous system NOS | F1z.. |  |
| *Demyelinating diseases of the central nervous system* | |  |
| Other central nervous system demyelinating diseases | F21.. |  |
| Neuromyelitis optica | F210. |  |
| Schilder's disease | F211. |  |
| Acute and subacute haemorrhagic leukoencephalitis [Hurst] | F212. |  |
| Clinically isolated syndrome | F213. |  |
| Acute disseminated demyelination, unspecified | F21X. |  |
| Other acute disseminated demyelination |  | G36 |
| Other specified central nervous system demyelinating disease | F21y. | G37 |
| Central nervous system demyelination NOS | F21z. |  |
| Marchiafava-Bignami disease | F21y0 |  |
| Central pontine myelinosis | F21y1 |  |
| Binswanger's disease | F21y2 |  |
| Central demyelination of corpus callosum | F21y3 |  |
| Subacute necrotizing myelitis | F21y4 |  |
| Concentric sclerosis | F21y5 |  |
| Vanishing white matter disease | F21y6 |  |
| Other specified central nervous system demyelination NOS | F21yz |  |
| Transverse myelitis | F037. |  |
| Varicella transverse myelitis | F0370 |  |
| Multiple sclerosis | F20.. | G35 |
| FH: Multiple sclerosis | 1292. |  |
| Multiple sclerosis NOS | F20z. |  |
| Multiple sclerosis of the brain stem | F200. |  |
| Multiple sclerosis of the spinal cord | F201. |  |
| Generalised multiple sclerosis | F202. |  |
| Exacerbation of multiple sclerosis | F203. |  |
| Benign multiple sclerosis | F204. |  |
| Malignant multiple sclerosis | F205. |  |
| Primary progressive multiple sclerosis | F206. |  |
| Relapsing and remitting multiple sclerosis | F207. |  |
| Secondary progressive multiple sclerosis | F208. |  |
| Niemann-Pick disease | C3272 |  |
| Progressive supranuclear palsy | F24y0 |  |
| Wilson's disease | C3510 |  |
| Multiple system atrophy | F174. |  |
| Multiple system atrophy, cerebellar variant | F1740 |  |
| Multiple system atrophy, Parkinson variant | F1741 |  |
| [X] Lewy body dementia | Eu025 |  |
| *Cerebral palsy* |  | G80 |
| Athetoid cerebral palsy | F1370 |  |
| Congenital cerebral palsy | F23.. |  |
| Congenital diplegia | F230. |  |
| Congenital paraplegia | F2300 |  |
| Cerebral palsy with spastic diplegia | F2301 |  |
| Congenital diplegia NOS | F230z |  |
| Congenital hemiplegia | F231. |  |
| Congenital quadriplegia | F232. |  |
| Congenital monoplegia | F233. |  |
| Infantile hemiplegia NOS | F234. |  |
| Other congenital cerebral palsy | F23y. |  |
| Ataxic infantile cerebral palsy | F23y0 |  |
| Flaccid infantile cerebral palsy | F23y1 |  |
| Spastic cerebral palsy | F23y2 |  |
| Dyskinetic cerebral palsy | F23y3 |  |
| Ataxic diplegic cerebral palsy | F23y4 |  |
| Choreoathetoid cerebral palsy | F23y6 |  |
| Other infantile cerebral palsy NOS | F23yz |  |
| Congenital cerebral palsy NOS | F23z. |  |
| FH: Infantile cerebral palsy | 1295. |  |
| Cerebral palsy, not congenital or infantile, acute | G669. |  |
| *Tics/tic disorders* |  | F95 |
| Gilles de la Tourette's disorder | E2723 |  |
| [X]Combined vocal and multiple motor tic disorder [de la Tourette] | Eu952 |  |
| Tic - symptom | 1B24. |  |
| O/E - spasm/tic | 2974. |  |
| Tic disorder unspecified | E2720 |  |
| Transiet childhood tic | E2721 |  |
| Chronic motor tic disorder | E2722 |  |
| Tic NOS | E272z |  |
| [X]Tic disorders | Eu95. |  |
| [X]Transient tic disorder | Eu950 |  |
| [X]Chronic motor or vocal tic disorder | Eu951 |  |
| [X]Involuntary excessive blinking | Eu953 |  |
| [X]Other tic disorders | Eu95y |  |
| [X]Tic disorder, unspecified | Eu95z |  |
| Tics | E272. |  |
| Tics of organic origin | F133. |  |
| *Brain tumour* |  |  |
| Neuroblastoma | B546. |  |
| [M]Neuroblastoma NOS | BBc1. |  |
| [M]Olfactory neuroblastoma | BBcC. |  |
| Benign neoplasm of brain and other parts of central nervous system |  | D33 |
| Benign neoplasm of brain | B7F0. |  |
| Benign neoplasm of brain, supratentorial | B7F00 |  |
| Malignant neoplasm of brain | B51.. | C71 |
| Malignant neoplasm of cerebrum (excluding lobes and ventricles) | B510. |  |
| Malignant neoplasm of basal ganglia | B5100 |  |
| Malignant neoplasm of cerebral cortex | B5101 |  |
| Malignant neoplasm of corpus striatum | B5102 |  |
| Malignant neoplasm of globus pallidus | B5103 |  |
| Malignant neoplasm of hypothalamus | B5104 |  |
| Malignant neoplasm of thalamus | B5105 |  |
| Malignant neoplasm of cerebrum NOS | B510z |  |
| Malignant neoplasm of frontal lobe | B511. |  |
| Malignant neoplasm of temporal lobe | B512. |  |
| Malignant neoplasm of hippocampus | B5120 |  |
| Malignant neoplasm of uncus | B5121 |  |
| Malignant neoplasm of temporal lobe NOS | B512z |  |
| Malignant neoplasm of parietal lobe | B513. |  |
| Malignant neoplasm of occipital lobe | B514. |  |
| Malignant neoplasm of cerebral ventricles | B515. |  |
| Malignant neoplasm of floor of cerebral ventricle | B5151 |  |
| Malignant neoplasm of cerebral ventricle NOS | B515z |  |
| Malignant neoplasm of cerebellum | B516. |  |
| Malignant neoplasm of brain stem | B517. |  |
| Malignant neoplasm of cerebral peduncle | B5170 |  |
| Malignant neoplasm of medulla oblongata | B5171 |  |
| Malignant neoplasm of midbrain | B5172 |  |
| Malignant neoplasm of pons | B5173 |  |
| Malignant neoplasm of brain stem NOS | B517z |  |
| Malignant neoplasm of other parts of brain | B51y. |  |
| Malignant neoplasm of corpus callosum | B51y0 |  |
| Malignant neoplasm of tapetum | B51y1 |  |
| Malignant neoplasm, overlapping lesion of brain | B51y2 |  |
| Malignant neoplasm of other part of brain NOS | B51yz |  |
| Malignant neoplasm of brain NOS | B51z. |  |
| Secondary malignant neoplasm of brain | B5830 |  |
| Neoplasm of unspecified nature of brain | BA06. |  |
| Neoplasm of uncertain behaviour of brain | B9250 |  |
| Neoplasm of uncertain or unknown behaviour of brain, infratentorial | B9253 |  |
| Malignant neoplasm, overlapping lesion of brain and other part of central nervous system | B52W. |  |
| Neoplasm of uncertain or unknown behaviour of brain, supratentorial | B9252 |  |
| [M]Astrocytoma NOS | BBbB. |  |
| [M]Pilocytic astrocytoma | BBbG. |  |
| [M]Fibrillary astrocytoma | BBbF. |  |
| [M]Gemistocytic astrocytoma | BBbE. |  |
| [M]Protoplasmic astrocytoma | BBbD. |  |
| [M]Astrocytoma, anaplastic type | BBbC. |  |
| [M]Subependymal astrocytoma NOS | BBb3. |  |
| [M]Subependymal giant cell astrocytoma | BBb4. |  |
| [M]Subependymoma | BBb3. |  |
| [M]Glioma NOS | BBb0. |  |
| [M]Gliomas | BBb.. |  |
| [M]Glioma, malignant | BBb0. |  |
| [M]Gliomatosis cerebri | BBb1. |  |
| [M]Mixed glioma | BBb2. |  |
| [M]Subependymal glioma | BBb3. |  |
| [M]Choroid plexus papilloma NOS | BBb5. |  |
| [M]Choroid plexus papilloma, malignant | BBb6. |  |
| [M]Ependymoma NOS | BBb7. |  |
| [M]Ependymoblastoma | BBb8. |  |
| [M]Ependymoma, anaplastic type | BBb8. |  |
| [M]Papillary ependymoma | BBb9. |  |
| [M]Myxopapillary ependymoma | BBbA. |  |
| [M]Spongioblastoma NOS | BBbH. |  |
| [M]Spongioblastoma polare | BBbJ. |  |
| [M]Glioblastoma NOS | BBbL. |  |
| [M]Giant cell glioblastoma | BBbM. |  |
| [M]Glioblastoma with sarcomatous component | BBbN. |  |
| [M]Primitive polar spongioblastoma | BBbP. |  |
| [M]Oligodendroglioma NOS | BBbQ. |  |
| [M]Oligodendroglioma, anaplastic type | BBbR. |  |
| [M]Oligodendroblastoma | BBbS. |  |
| [M]Medulloblastoma NOS | BBbT. |  |
| [M]Desmoplastic medulloblastoma | BBbU. |  |
| [M]Medullomyoblastoma | BBbV. |  |
| [M]Cerebellar sarcoma NOS | BBbW. |  |
| [M]Monstrocellular sarcoma | BBbX. |  |
| [M]Pleomorphic xanthoastrocytoma | BBbZ. |  |
| [M]Primitive neuroectodermal tumour | BBba. |  |
| [M]Peripheral neuroectodermal tumour | BBba0 |  |
| [M]Glioma NOS | BBbz. |  |
| Cerebral meningioma | B7F20 |  |
| Benign neoplasm of pineal gland | B7H3. |  |
| Malignant neoplasm of pineal gland | B543. |  |
| Neoplasm of uncertain behaviour of pineal gland | B921. |  |
| [M]Pinealoma | BBa1. |  |
| Malignant neoplasm of olfactory bulb | B5200 |  |
| [M]Olfactory neuroepithelioma | BBcD. |  |
| [M]Olfactory neurogenic tumour | BBcA. |  |
| Pituitary adenoma | B7H2. |  |
| Benign neoplasm of pituitary gland | B7H20 |  |
| Benign neoplasm of Rathke's pouch | B7H21 |  |
| Benign neoplasm of sella turcica | B7H22 |  |
| Benign neoplasm of craniopharyngeal duct | B7H23 |  |
| Benign neoplasm of pituitary gland and craniopharyngeal duct NOS | B7H2z |  |
| Malignant neoplasm of pituitary gland | B5420 |  |
| [M]Pituitary adenomas and carcinomas | BB5V. |  |
| [M]Prolactinoma | BB5y4 |  |
| [M]Pituitary adenoma or carcinoma NOS | BB5Vz |  |
| Cerebral metastasis | B5832 |  |
| Secondary malignant neoplasm of brain or spinal cord NOS | B583z |  |
| [M]Craniopharyngioma | BBa0. |  |
| Benign neoplasm of craniopharyngeal duct | B7H23 |  |
| Malignant neoplasm of craniopharyngeal duct | B5421 |  |
| Neoplasm of uncertain behaviour of craniopharyngeal duct | B9201 |  |
| [M]Chordoma | BBa5. |  |
| [M]Schwannoma NOS | BBe5. |  |
| [M]Schwannoma, malignant | BBe7. |  |
| Acoustic neuroma | B7F10 |  |
| [M]Primitive neuroectodermal tumour | BBba. |  |
| [M]Haemangioblastic meningioma | BBd7. |  |
| [M]Meningiomas | BBd.. |  |
| [M]Meningioma NOS | BBd0. |  |
| [M]Meningiomatosis NOS | BBd1. |  |
| [M]Meningioma, malignant | BBd2. |  |
| [M]Meningotheliomatous meningioma | BBd3. |  |
| [M]Fibrous meningioma | BBd4. |  |
| [M]Psammomatous meningioma | BBd5. |  |
| [M]Angiomatous meningioma | BBd6. |  |
| [M]Haemangiopericytic meningioma | BBd8. |  |
| [M]Transitional meningioma | BBd9. |  |
| [M]Papillary meningioma | BBdA. |  |
| [M]Meningeal sarcomatosis | BBdB. |  |
| [M]Meningioma NOS | BBdz. |  |
| Spinal meningioma | B7F40 |  |
| *Metabolic disorders* |  |  |
| Disorders of aromatic amino-acid metabolism |  | E70 |
| Other disturbances of aromatic amino-acid metabolism | C302. |  |
| Alkaptonuria | C3020 |  |
| Hydroxykynureninuria | C3021 |  |
| Indicanuria | C3022 |  |
| Tyrosinosis | C3023 |  |
| Tyrosinuria | C3024 |  |
| Lowe disease | C3025 |  |
| Hypertyrosinaemia | C3026 |  |
| Albinism | C3027 |  |
| Ocular albinism | P3y0. |  |
| Chediak-Higashi syndrome | C3028 |  |
| Hermansky-Pudlak syndrome | C3029 |  |
| Partial albinism | C302A |  |
| Other specified disturbance of aromatic amino-acid metabolism | C302y |  |
| Disturbance of aromatic amino-acid metabolism NOS | C302z |  |
| Phenylketonuria | C301. |  |
| Disturbance of histidine metabolism | C305. |  |
| Histidinaemia | C3050 |  |
| Imidazole aminoaciduria | C3051 |  |
| Histidinuria | C3052 |  |
| Other specified disturbance of histidine metabolism | C305y |  |
| Disturbance of histidine metabolism NOS | C305z |  |
| Disorders of fatty-acid metabolism | C308. |  |
| Medium chain acyl-CoA dehydrogenase deficiency | C3080 |  |
| Multiple acyl-CoA dehydrogenase deficiencies | C3081 |  |
| X-linked adrenoleucodystrophy | C3082 |  |
| Glutaryl CoA dehydrogenase deficiency | C309. |  |
| Tryptophan malabsorption syndrome | C30A. |  |
| Disorders of branched-chain amino-acid metabolism and fatty-acid metabolism |  | E71 |
| Disturbances of branched-chain amino-acid metabolism | C303. |  |
| Leucinosis | C3030 |  |
| Isoleucinosis | C3031 |  |
| Hypervalinaemia | C3032 |  |
| Maple syrup urine disease | C3033 |  |
| Hypervalinaemia | C3034 |  |
| Other specified disturbance of branched chain amino-acid metabolism | C303y |  |
| Disturbance of branched-chain amino-acid metabolism NOS | C303z |  |
| Disorders of amino-acid transport | C300. |  |
| Cystinosis | C3000 |  |
| Cystinuria | C3001 |  |
| Fanconi-de-Toni syndrome | C3003 |  |
| Hartnup disease | C3004 |  |
| Succinic semialdehyde dehydrogenase deficiency | C3005 |  |
| Acquired Fanconi syndrome | C3006 |  |
| Adult Fanconi syndrome | C3007 |  |
| Juvenile nephropathic cystinosis | C3008 |  |
| Adult cystinosis | C3009 |  |
| Congenital Fanconi syndrome | C300A |  |
| Dibasic aminoaciduria - type I | C300B |  |
| Lysinuric protein intolerance | C300C |  |
| Infantile nephropathic cystinosis | C300D |  |
| Dibasic aminoaciduria | C300E |  |
| Other specified amino-acid transport disorder | C300y |  |
| Amino-acid transport disorder NOS | C300z |  |
| Glutaric aciduria Type 1 | C30y8 |  |
| Homocystinuria | C3043 |  |
| Disturbance of urea cycle metabolism | C306. |  |
| Hyperornithinaemia | C3060 |  |
| Citrullinaemia | C3061 |  |
| Argininosuccinic aciduria | C3062 |  |
| Hyperargininaemia | C3063 |  |
| Hyperammonaemia | C3064 |  |
| Other specified disturbance of urea cycle metabolism | C306y |  |
| Disturbance of urea cycle metabolism NOS | C306z |  |
| Disturbance of sulphur-bearing amino-acid metabolism | C304. |  |
| Cystathioninaemia | C3040 |  |
| Cystathioninuria | C3041 |  |
| Methioninaemia | C3042 |  |
| Homocystinuria | C3043 |  |
| Sulphite oxidase deficiency | C3044 |  |
| Hyperhomocysteinaemia | C3045 |  |
| Other specified disturbance of sulphur-bearing amino-acid metabolism | C304y |  |
| Disturbance of sulphur-bearing amino-acid metabolism NOS | C304z |  |
| Disturbance of other straight-chain amino-acid metabolism | C307. |  |
| Hyperglycinaemia | C3070 |  |
| Disturbance of threonine metabolism | C3071 |  |
| Disturbance of serine metabolism | C3072 |  |
| Disturbance of glutamine metabolism | C3073 |  |
| Hyperlysinaemia | C3074 |  |
| Pipecolic acidaemia | C3075 |  |
| Saccharopinuria | C3076 |  |
| Glucoglycinuria | C3077 |  |
| Other specified disturbance of other straight-chain amino-acid metabolism | C307y |  |
| Disturbance of other straight-chain amino-acid metabolism NOS | C307z |  |
| Disturbance of other specified amino-acid metabolism | C30y. |  |
| Alaninaemia | C30y0 |  |
| Ethanolaminuria | C30y1 |  |
| Glycoprolinuria | C30y2 |  |
| Hydroxyprolinaemia | C30y3 |  |
| Hyperprolinaemia | C30y4 |  |
| Prolinuria | C30y5 |  |
| Iminoacidopathy | C30y6 |  |
| Sarcosinaemia | C30y7 |  |
| Other specified disturbance of amino-acid metabolism | C30yy |  |
| Disturbance of other specified amino-acid metabolism NOS | C30yz |  |
| Other disorders of amino-acid metabolism |  | E72 |
| Disturbance of amino-acid transport or metabolism NOS | C30z. |  |
| Disorders of carbohydrate transport and metabolism | C31.. |  |
| Other disorders of carbohydrate metabolism |  | E74 |
| Glycogenosis - glycogen storage disease | C310. |  |
| McArdle's disease | C3100 |  |
| Generalised glycogenosis | C3101 |  |
| Hepatorenal glycogenosis | C3102 |  |
| Glycogenosis of liver and muscle | C3103 |  |
| Glycogenosis with hepatic cirrhosis | C3104 |  |
| Other specified glycogenosis | C310y |  |
| Glycogenosis NOS | C310z |  |
| Galactosaemia | C311. |  |
| Galactose-1-phosphate uridyl transferase deficiency | C3110 |  |
| Galactokinase deficiency | C3111 |  |
| Other specified galactosaemia | C311y |  |
| Galactosaemia NOS | C311z |  |
| Hereditary fructose intolerance | C312. |  |
| Lactase deficiency | C3137 |  |
| Lactose intolerance | C3131 | E73 |
| Primary lactose intolerance | C3133 |  |
| Secondary lactose intolerance | C3134 |  |
| Acquired fructose intolerance | C316. |  |
| Disorders of pyruvate metabolism and gluconeogenesis | C315. |  |
| Pyruvate dehydrogenase deficiency | C3150 |  |
| Mitochondrial encephalopathy, lactic acidosis and stroke-like episodes | C3151 |  |
| Kearns-Sayre syndrome | C3152 |  |
| Other disorders of carbohydrate transport and metabolism | C31y. |  |
| Essential benign pentosuria | C31y0 |  |
| Fucosidosis | C31y1 |  |
| Oxalosis | C31y2 |  |
| Mannosidosis | C31y3 |  |
| Xylosuria | C31y4 |  |
| Primary oxaluria | C31y5 |  |
| Oxaluria NEC | C31y6 |  |
| Aspartylglucosaminuria | C31y7 |  |
| Glycerol kinase deficiency | C31y8 |  |
| Disorder of glycoprotein metabolism, unspecified | C31yX |  |
| Other disorders of carbohydrate transport and metabolism NOS | C31yz |  |
| Disorder of carbohydrate transport or metabolism NOS | C31z. |  |
| Disorders of lipoid metabolism | C32.. |  |
| Pure hypercholesterolaemia | C320. |  |
| Familial hypercholesterolaemia | C3200 |  |
| Hyperbetalipoproteinaemia | C3201 |  |
| Hyperlipidaemia, group A | C3202 |  |
| Low-density-lipoprotein-type (LDL) hyperlipoproteinaemia | C3203 |  |
| Fredrickson's hyperlipoproteinaemia, type IIa | C3204 |  |
| Familial defective apolipoprotein B-100 | C3205 |  |
| Polygenic hypercholesterolaemia | C3206 |  |
| Other specified pure hypercholesterolaemia | C320y |  |
| Pure hypercholesterolaemia NOS | C320z |  |
| Pure hyperglyceridaemia | C321. |  |
| Hypertriglyceridaemia | C3210 |  |
| Mixed hyperlipidaemia | C322. |  |
| Familial combined hyperlipidaemia | C3220 |  |
| Hyperchylomicronaemia | C323. |  |
| Hyperlipidaemia NOS | C324. |  |
| Lipoprotein deficiencies | C325. |  |
| High density lipoid deficiency | C3250 |  |
| Hypo-alpha-lipoproteinaemia | C3251 |  |
| Hypo-beta-lipoproteinaemia | C3252 |  |
| A-beta-lipoproteinaemia | C3253 |  |
| Lipoprotein deficiency NOS | C325z |  |
| Lipodystrophy | C326. |  |
| Progressive lipodystrophy | C3260 |  |
| Lipodystrophy NOS | C326z |  |
| Lipidoses | C327. |  |
| Chemically induced lipidosis | C3270 |  |
| Krabbe's disease | F1000 |  |
| Gaucher's disease | C3271 |  |
| Niemann-Pick disease | C3272 |  |
| Wolman disease | C3273 |  |
| Alpha-galactosidase A deficiency | C3274 |  |
| Lipidoses NOS | C327z |  |
| Dyslipidaemia | C328. |  |
| Hypercholesterolaemia | C329. |  |
| Other disorders of lipoid metabolism | C32y. |  |
| Liposynovitis prepatellaris | C32y0 |  |
| Launois-Bensaude's lipomatosis | C32y1 |  |
| Lipoid dermatoarthritis | C32y2 |  |
| Pelvic lipomatosis | C32y3 |  |
| Lipase deficiency | C32y4 |  |
| Steatosis | C32y5 |  |
| Lipomatosis NEC | C32y6 |  |
| Other disorder of lipoid metabolism NOS | C32yz |  |
| Disorders of lipoprotein metabolism and other lipidemias |  | E78 |
| Disorders of sphingolipid metabolism and other lipid storage disorders |  | E75 |
| Disorders of mineral metabolism | C35.. | E83 |
| Disorders of iron metabolism | C350. |  |
| Haemochromatosis | C3500 |  |
| Haemosiderosis, primary | C3501 |  |
| Haemosiderosis, acquired | C3502 |  |
| Idiopathic pulmonary haemosiderosis | C3503 |  |
| Haemosiderosis NOS | C3504 |  |
| Other specified disorder of iron metabolism | C350y |  |
| Disorder of iron metabolism NOS | C350z |  |
| Disorders of copper metabolism | C351. |  |
| Hepatolenticular degeneration (Wilson's disease) | C3510 |  |
| Hypercupraemia | C3511 |  |
| Disorder of copper metabolism NOS | C351z |  |
| Disorders of magnesium metabolism | C352. |  |
| Hypermagnesaemia | C3520 |  |
| Hypomagnesaemia | C3521 |  |
| Hypomagnesaemic tetany | C3522 |  |
| Disorder of magnesium metabolism NOS | C352z |  |
| Disorders of phosphorus metabolism | C353. |  |
| Hypophosphatasia | C3530 |  |
| Hypophosphatasia rickets | C3531 |  |
| Vitamin-D-resistant rickets | C3532 |  |
| Hypophosphataemia | C3533 |  |
| Hyperphosphataemia | C3534 |  |
| Acquired hypophosphataemia | C3535 |  |
| Renal failure-associated hyperphosphataemia | C3536 |  |
| X-linked hypophosphataemic rickets | C3537 |  |
| Autosomal dominant hypophosphataemic rickets | C3538 |  |
| Autosomal recessive hypophosphataemic rickets | C3539 |  |
| Disorder of phosphorus metabolism NOS | C353z |  |
| Disorders of calcium metabolism | C354. |  |
| Hypocalcaemia NEC | C3540 |  |
| Hypercalcaemia NEC | C3541 |  |
| Idiopathic hypercalcaemia | C3542 |  |
| Pseudohypoparathyroidism | C3543 |  |
| Hypercalcinuria | C3544 |  |
| Familial benign hypercalcaemia | C3545 |  |
| Hypocalcaemic tetany | C3546 |  |
| Nephrocalcinosis | C3547 |  |
| Other calcinosis | C3548 |  |
| Calcium deficiency | C3549 |  |
| Metastatic calcification | C354A |  |
| Calciphylaxis | C354B |  |
| Cortical nephrocalcinosis | C354C |  |
| Familial hypocalciuric hypercalcaemia | C354D |  |
| Other specified disorder of calcium metabolism | C354y |  |
| Disorder of calcium metabolism NOS | C354z |  |
| Disorders of zinc metabolism | C355. |  |
| Acrodermatitis enteropathica | C3550 |  |
| Disorders of phosphatases | C356. |  |
| Disorder of other specified mineral metabolism | C35y. |  |
| Disorder of mineral metabolism NOS | C35z. |  |
| Disorders of bilirubin excretion | C374. |  |
| Crigler - Najjar syndrome | C3740 |  |
| Dubin - Johnson syndrome | C3741 |  |
| Gilbert's syndrome | C3742 |  |
| Rotor syndrome | C3743 |  |
| Other specified congenital hyperbilirubinaemia | C374y |  |
| Congenital hyperbilirubinaemia NOS | C374z |  |
| Mucopolysaccharidosis | C375. |  |
| Mannosidosis | C3750 |  |
| Mucopolysaccharidosis, type 1 | C3751 |  |
| Mucopolysaccharidosis, type II | C3752 |  |
| Mucopolysaccharidosis, type III | C3753 |  |
| Mucopolysaccharidosis, type IV | C3754 |  |
| Pyknodysostosis | C3755 |  |
| Mucopolysaccharidosis, type VI | C3756 |  |
| Mucopolysaccharidosis, type VII | C3757 |  |
| Multiple sulphatase deficiency | C3758 |  |
| Disorders of glycosaminoglycan metabolism |  | E76 |
| Disorder of glucosaminoglycan metabolism, unspecified | C375X |  |
| Other specified mucopolysaccharidosis | C375y |  |
| Mucopolysaccharidosis NOS | C375z |  |
| Disorders of glycoprotein metabolism | C377. | E77 |
| Defects in post-translational modification of lysosomal enzymes | C3770 |  |
| Mucolipidosis type III | C3771 |  |
| Other disorders of purine and pyrimidine metabolism | C372. |  |
| Disorders of purine and pyrimidine metabolism |  | E79 |
| Hypoxanthine-guanine-phosphoribosyltransferase deficiency | C3720 |  |
| Xanthinuria | C3721 |  |
| Hyperuricaemia without signs of inflammatory arthritis and tophaceous disease | C3722 |  |
| Lesch-Nyhan syndrome | C3723 |  |
| Urate nephropathy | C3724 |  |
| Acute urate nephropathy | C3725 |  |
| Other disorder of purine or pyrimidine metabolism NOS | C372z |  |
| Hyperuricaemia without signs of inflammatory arthritis and tophaceous disease | C3722 |  |
| Gangliosidosis | F1014 |  |
| Lipofuscinosis NEC | F427K |  |
| Lipofuscinosis NOS | F427K |  |
| Cerebral degeneration in mucopolysaccharidoses | F1031 |  |
| Disorders of porphyrin metabolism | C371. |  |
| Disorders of porphyrin and bilirubin metabolism |  | E80 |
| Congenital porphyria | C3710 |  |
| Erythropoietic protoporphyria | C3711 |  |
| Acute intermittent porphyria | C3712 |  |
| Protocoproporphyria | C3713 |  |
| Porphyria cutanea tarda | C3714 |  |
| Coproporphyria | C3715 |  |
| Pseudoporphyria | C3716 |  |
| Porphyria NOS | C371z |  |
| Other deficiencies of circulating enzymes | C376. |  |
| Alpha-1-antitrypsin deficiency | C3762 |  |
| Other specified circulating enzyme deficiency | C376y |  |
| Alpha-1-antitrypsin hepatitis | C3761 |  |
| Deficiency of circulating enzyme NOS | C376z |  |
| Plasma protein metabolism disorders | C33.. |  |
| Polyclonal hypergammaglobulinaemia | C330. |  |
| Waldenstrom's hypergammaglobulinaemic purpura | C3300 |  |
| Benign primary hypergammaglobulinaemic purpura | C3301 |  |
| Polyclonal hypergammaglobulinaemia NOS | C330z |  |
| Monoclonal paraproteinaemia | C331. |  |
| Other paraproteinaemias | C332. |  |
| Cryoglobulinaemic purpura | C3320 |  |
| Cryoglobulinaemic vasculitis | C3321 |  |
| Benign paraproteinaemia | C3322 |  |
| Paraproteinaemia NOS | C332z |  |
| Macroglobulinaemia | C333. |  |
| Waldenstrom's macroglobulinaemia | C3330 |  |
| Alpha heavy chain disease | C3331 |  |
| Gamma heavy chain disease | C3332 |  |
| Heavy chain disease | C3333 |  |
| Macroglobulinaemia NOS | C333z |  |
| Amyloidosis |  | E85 |
| Other disorder of plasma protein metabolism | C33y. |  |
| Hypoproteinaemia | C33y0 |  |
| Other specified other disorders of plasma protein metabolism | C33yy |  |
| Other disorders of plasma protein metabolism NOS | C33yz |  |
| Disorder of plasma protein metabolism NOS | C33z. |  |
| Tumour lysis syndrome | C37yD |  |
| Metabolic syndrome | C1A0. |  |
| Smith - Lemli - Opitz syndrome | PKy63 |  |
| Lipoid dermatoarthritis | C32y2 |  |
| *Acute rheumatic fever* |  |  |
| Acute rheumatic fever | G0... |  |
| Rheumatic fever without heart involvement | G00.. |  |
| Rheumatic fever with heart involvement | G01.. |  |
| Acute rheumatic pericarditis | G010. |  |
| Acute rheumatic endocarditis | G011. |  |
| Acute rheumatic myocarditis | G012. |  |
| Other acute rheumatic heart disease | G01y. |  |
| Acute rheumatic pancarditis | G01y0 |  |
| Other acute rheumatic heart disease NOS | G01yz |  |
| Rheumatic chorea | G02.. | I02 |
| Rheumatic chorea with heart involvement | G020. | I01 |
| Rheumatic chorea without mention of heart involvement | G021. | I00 |
| Rheumatic chorea NOS | G02z. |  |
| Other specified acute rheumatic fever | G0y.. |  |
| Acute rheumatic fever NOS | G0z.. |  |
| *Congenital malformations of the central nervous system* |  |  |
| Anencephaly and similar malformations | Q00 |  |
| Anencephalus | P00.. |  |
| Acrania | P000. |  |
| Amyelencephalus | P001. |  |
| Hemicephaly | P002. |  |
| Other specified anencephalus | P00y. |  |
| Anencephalus NOS | P00z. |  |
| Craniorachischisis | P01.. |  |
| Iniencephaly | P02.. |  |
| Iniencephaly - closed | P020. |  |
| Open iniencephaly | P021. |  |
| Iniencephaly NOS | P02z. |  |
| Encephalocele | P20.. |  |
| Meningoencephalocele | P204. |  |
| Nasofrontal encephalocele | P206. |  |
| Frontal encephalocele | P205. |  |
| Meningocele - cerebral | P203. |  |
| Encephalocystocele | P200. |  |
| Encephalomyelocele | P201. |  |
| Hydromeningocele - cranial | P202. |  |
| Occipital encephalocele | P20z0 |  |
| Encephalocele of other specified site | P20z1 |  |
| Encephalocele NOS | P20z. |  |
| Encephalocele |  | Q01 |
| Microcephalus | P21.. |  |
| Microcephaly |  | Q02 |
| Hydromicrocephaly | P210. |  |
| Micrencephaly | P211. |  |
| Microcephalus NOS | P21z. |  |
| Congenital hydrocephalus | P23.. | Q03 |
| Aqueduct of Sylvius anomaly | P230. |  |
| Aqueduct of Sylvius obstruction | P2300 |  |
| Aqueduct of Sylvius stenosis | P2301 |  |
| Atresia of aqueduct of Sylvius NEC | P2302 |  |
| Aqueduct of Sylvius anomaly NOS | P230z |  |
| Foramen of Magendie atresia | P231. |  |
| Foramen of Luschka atresia | P232. |  |
| Atresia of foramina of Magendie and Luschka | P233. |  |
| Hydranencephaly | P234. |  |
| X-linked hydrocephalus | P235. |  |
| Other specified congenital hydrocephalus | P23y. |  |
| Congenital hydrocephalus NOS | P23z. |  |
| Congenital absence of corpus callosum | P2280 |  |
| Aicardi syndrome | P2283 |  |
| Anomaly of corpus callosum NOS | P228z |  |
| Aplasia of corpus callosum | P2282 |  |
| Hypoplasia of corpus callosum | P2281 |  |
| Anomalies of corpus callosum | P228. |  |
| Arhinencephaly | P224. |  |
| Septo-optic dysplasia | P246. |  |
| Megalencephaly | P249. |  |
| Congenital cerebral cyst | P240. |  |
| Single congenital cerebral cyst | P2400 |  |
| Multiple congenital cerebral cysts | P2401 |  |
| Schizencephaly | P2402 |  |
| Congenital cerebral cyst NOS | P240z |  |
| Other nervous system congenital anomalies | P2... |  |
| Macroencephaly | P241. |  |
| Macrogyria | P242. |  |
| Porencephaly | P243. |  |
| Ulegyria | P244. |  |
| Congenital adhesions of cerebral meninges | P245. |  |
| Dysplasia of cerebral cortex | P247. |  |
| Congenital dilated lateral ventricles of brain | P248. |  |
| Hemimegalencephaly | P24A. |  |
| Multiple brain anomalies | P24x. |  |
| Other specified brain anomalies NOS | P24z. |  |
| Spina bifida | P1... | Q05 |
| Spina bifida with hydrocephalus | P10.. |  |
| Unspecified spina bifida with hydrocephalus | P100. |  |
| Spina bifida with hydrocephalus, unspecified | P1000 |  |
| Cervical spina bifida with hydrocephalus | P1001 |  |
| Thoracic spina bifida with hydrocephalus | P1002 |  |
| Lumbar spina bifida with hydrocephalus | P1003 |  |
| Spina bifida with hydrocephalus NOS | P100z |  |
| Arnold - Chiari syndrome | P101. |  |
| Chiari malformation type I | P1010 |  |
| Chiari malformation type II | P1011 |  |
| Chiari malformation type III | P1012 |  |
| Chiari malformation type IV | P1013 |  |
| Spina bifida with hydrocephalus of late onset | P104. |  |
| Spina bifida with stenosis of aqueduct of Sylvius | P105. |  |
| Spina bifida with hydrocephalus - open | P102. |  |
| Unspecified spina bifida with hydrocephalus - open | P1020 |  |
| Cervical spina bifida with hydrocephalus - open | P1021 |  |
| Thoracic spina bifida with hydrocephalus - open | P1022 |  |
| Lumbar spina bifida with hydrocephalus - open | P1023 |  |
| Sacral spina bifida with hydrocephalus - open | P1024 |  |
| Spina bifida with hydrocephalus - open NOS | P102z |  |
| Spina bifida with hydrocephalus - closed | P103. |  |
| Unspecified spina bifida with hydrocephalus - closed | P1030 |  |
| Cervical spina bifida with hydrocephalus - closed | P1031 |  |
| Thoracic spina bifida with hydrocephalus - closed | P1032 |  |
| Lumbar spina bifida with hydrocephalus - closed | P1033 |  |
| Sacral spina bifida with hydrocephalus - closed | P1034 |  |
| Spina bifida with hydrocephalus - closed NOS | P103z |  |
| Other specified spina bifida with hydrocephalus | P10y. |  |
| Dandy - Walker syndrome with spina bifida | P10y0 |  |
| Other spina bifida with hydrocephalus NOS | P10yz |  |
| Spina bifida with hydrocephalus NOS | P10z. |  |
| Spina bifida without mention of hydrocephalus | P11.. |  |
| Spina bifida without mention of hydrocephalus, unspecified | P110. |  |
| Spina bifida without mention of hydrocephalus, site unspecified | P1100 |  |
| Cervical spina bifida without mention of hydrocephalus | P1101 |  |
| Thoracic spina bifida without mention of hydrocephalus | P1102 |  |
| Lumbar spina bifida without mention of hydrocephalus | P1103 |  |
| Unspecified spina bifida without mention of hydrocephalus NOS | P110z |  |
| Spina bifida NOS | P1z.. |  |
| Amyelia | P25y0 |  |
| Other specified spinal cord anomalies | P25.. |  |
| Diastematomyelia | P250. |  |
| Hydromyelia | P251. |  |
| Congenital tethering of spinal cord | P252. |  |
| Spinal cord anomalies NOS | P25z. |  |
| Atelomyelia | P25y1 |  |
| Congenital anomaly of spinal meninges | P25y2 |  |
| Defective development of the cauda equina | P25y3 |  |
| Spinal cord hypoplasia | P25y4 |  |
| Other specified spinal cord anomalies NOS | P25yz |  |
| Reduction deformities of brain | P22.. |  |
| Hypoplasia of brain, part unspecified | P222. |  |
| Agyria | P223. |  |
| Microgyria | P226. |  |
| Congenital bilateral perisylvian syndrome | P2260 |  |
| Holoprosencephaly | P225. |  |
| Anomalies of cerebrum | P227. |  |
| Congenital hypoplasia of cerebrum | P2271 |  |
| Anomaly of cerebrum NOS | P227z |  |
| Anomalies of hypothalamus | P229. |  |
| Anomalies of cerebellum | P22A. |  |
| Congenital absence of cerebellum | P22A0 |  |
| Hypoplasia of cerebellum | P22A1 |  |
| Aplasia of cerebellum | P22A2 |  |
| Anomaly of cerebellum NOS | P22Az |  |
| Other specified reduction deformities of brain | P22y. |  |
| Cebocephaly | P22y0 |  |
| Familial aplasia of the vermis | P22y1 |  |
| Gillespie syndrome | P22y2 |  |
| Partial absence of septum pellucidum | P22y3 |  |
| Other reduction deformity of brain NOS | P22yz |  |
| Reduction deformities of brain NOS | P22z. |  |
| Marcus - Gunn syndrome | P2x4. |  |
| Agenesis of nerve, unspecified | P2x0. |  |
| Gillespie syndrome | P22y2 |  |
| Structural central nervous system abnormality | P2x8. |  |
| Other specified nervous system anomalies NOS | P2xz. |  |
| Unspecified nervous system anomaly of brain, cord and nervous system | P2y.. |  |
| Congenital brain anomaly | P2y0. |  |
| Congenital spinal cord anomaly | P2y1. |  |
| Unspecified nervous system anomaly NOS | P2yz. |  |
| Other congenital malformations of brain |  | Q04 |
| Other congenital malformations of spinal cord |  | Q06 |
| Other congenital malformations of nervous system |  | Q07 |

**Key:** CNS; Central Nervous System, EC; Elsewhere Classified, FH; Family History, NEC; Not Elsewhere Classified, NOS; Not Otherwise Specified, O/E; On Examination, OS; Other Specified
