# Supplementary material for: Adult‐onset idiopathic dystonia: A national data‐linkage study to determine epidemiological, social deprivation, and mortality characteristics
Source: Eur J Neurol. 2021 Oct 15;29(1):91–104. doi: 10.1111/ene.15114 (PMC9377012; doi:10.1111/ene.15114)
Supplement: Supplementary file 3 [file ENE-29-91-s004.docx]

**Supplementary Table 3. Parkinson’s medication Read Codes used to exclude those with co-morbid dystonia diagnoses**

| **Parkinson’s medication** | **Read code** |
| --- | --- |
| AMANTADINE HYDROCHLORIDE 50mg/5mL syrup | dq44. |
| AMANTADINE HYDROCHLORIDE 100mg capsules | dq4z. |
| SYMMETREL [PARK] 100mg capsules | dq41. |
| SYMMETREL [PARK] 50mg/5mL syrup | dq42. |
| *MANTADINE 100mg capsules | dq43. |
| *PARLODEL [PARK] 1mg tablets | dq51. |
| *PARLODEL [PARK] 2.5mg tablets | dq52. |
| PARLODEL [PARK] 5mg capsules | dq53. |
| PARLODEL [PARK] 10mg capsules | dq54. |
| *PARLODEL STARTER | dq55. |
| BROMOCRIPTINE [PARK] 1mg tablets | dq56. |
| BROMOCRIPTINE [PARK] 5mg capsules | dq57. |
| BROMOCRIPTINE 2.5mg tablets | dq5y. |
| BROMOCRIPTINE 10mg capsules | dq5z. |
| ELDEPRYL 5mg tablets | dq61. |
| ELDEPRYL 10mg tablets | dq62. |
| *ELDEPRYL 10mg/5mL syrup | dq63. |
| *VIVAPRYL 5mg tablets | dq64. |
| *VIVAPRYL 10mg tablets | dq65. |
| *STILLINE 5mg tablets | dq66. |
| *STILLINE 10mg tablets | dq67. |
| *CENTRAPRYL 5 tablets | dq68. |
| *CENTRAPRYL 10 tablets | dq69. |
| *CENTRAPRYL 10 tablets | dq69. |
| ZELAPAR 1.25mg tablets | dq6A. |
| SELEGILINE HYDROCHLORIDE 1.25mg tablets | dq6w. |
| SELEGILINE HYDROCHLORIDE 10mg/5mL syrup | dq6x. |
| SELEGILINE HYDROCHLORIDE 10mg tablets | dq6y. |
| SELEGILINE HYDROCHLORIDE 5mg tablets | dq6z. |
| LISURIDE 200micrograms tablets | dq71. |
| *REVANIL 200micrograms tablets | dq72. |
| PERGOLIDE 50micrograms tablets | dq81. |
| PERGOLIDE 250micrograms tablets | dq82. |
| PERGOLIDE 1mg tablets | dq83. |
| PERGOLIDE 50micrograms+250micrograms tablets starter pack | dq89. |
| PERGOLIDE 50micrograms tablets starter pack | dq88. |
| *CELANCE 50micrograms tablets | dq84. |
| *CELANCE 250micrograms tablets | dq85. |
| *CELANCE 1mg tablets | dq86. |
| CELANCE 50micrograms tablets starter pack | dq87. |
| CELANCE 50micrograms+250micrograms tablets starter pack | dq8A. |
| *BRITAJECT 20mg/2mL injection | dq91. |
| APOMORPHINE HYDROCHLORIDE 20mg/2mL injection | dq92. |
| *BRITAJECT 50mg/5mL injection | dq93. |
| APOMORPHINE HYDROCHLORIDE 50mg/5mL injection | dq94. |
| APOMORPHINE HYDROCHLORIDE 30mg/3mL prefilled pen | dq95. |
| BRITAJECT 30mg/3mL prefilled pen | dq96. |
| APO-GO 20mg/2mL injection | dq97. |
| APO-GO 50mg/5mL injection | dq98. |
| APO-GO 30mg/3mL prefilled pen | dq99. |
| APO-GO PFS 50mg/10mL injection solution prefilled syringe | dq9A. |
| APOMORPHINE HYDROCHLORIDE 50mg/10mL prefilled syringe | dq9z. |
| ROPINIROLE 0.25mg tablets | dqA1. |
| ROPINIROLE 1mg tablets | dqA2. |
| ROPINIROLE 2mg tablets | dqA3. |
| ROPINIROLE 5mg tablets | dqA4. |
| *REQUIP 0.25mg tablets | dqA5. |
| REQUIP 1mg tablets | dqA6. |
| REQUIP 2mg tablets | dqA7. |
| REQUIP 5mg tablets | dqA8. |
| ROPINIROLE 250micrograms+500micrograms+1000micrograms tablets starter pack | dqA9. |
| EPPINIX XL 3mg m/r tablets | dqAA. |
| EPPINIX XL 4mg m/r tablets | dqAB. |
| EPPINIX XL 6mg m/r tablets | dqAC. |
| EPPINIX XL 8mg m/r tablets | dqAD. |
| ROPINIROLE 500micrograms+1mg+2mg tablets follow on pack | dqAa. |
| REQUIP tablets starter pack | dqAb. |
| REQUIP tablets follow-on pack | dqAc. |
| ADARTREL 2mg tablets | dqAd. |
| ROPINIROLE 500micrograms tablets | dqAe. |
| ADARTREL 500micrograms tablets | dqAf. |
| REQUIP XL 2mg m/r tablets | dqAg. |
| REQUIP XL 4mg m/r tablets | dqAh. |
| REQUIP XL 8mg m/r tablets | dqAi. |
| ROPINIROLE 2mg m/r tablets | dqAj. |
| ROPINIROLE 4mg m/r tablets | dqAk. |
| ROPINIROLE 8mg m/r tablets | dqAl. |
| REPINEX XL 2mg m/r tablets | dqAm. |
| REPINEX XL 4mg m/r tablets | dqAn. |
| REPINEX XL 8mg m/r tablets | dqAo. |
| RAPONER XL 2mg m/r tablets | dqAp. |
| RAPONER XL 3mg m/r tablets | dqAq. |
| ROPINIROLE 3mg m/r tablets | dqAr. |
| RAPONER XL 4mg m/r tablets | dqAs. |
| RAPONER XL 6mg m/r tablets | dqAt. |
| ROPINIROLE 6mg m/r tablets | dqAu. |
| RAPONER XL 8mg m/r tablets | dqAv. |
| AIMPART XL 2mg m/r tablets | dqAw. |
| AIMPART XL 4mg m/r tablets | dqAx. |
| AIMPART XL 8mg m/r tablets | dqAy. |
| EPPINIX XL 2mg m/r tablets | dqAz. |
| CABERGOLINE 1mg tablets | dqB1. |
| CABERGOLINE 2mg tablets | dqB2. |
| CABERGOLINE 4mg tablets | dqB3. |
| CABASER 1mg tablets | dqB4. |
| CABASER 2mg tablets | dqB5. |
| *CABASER 4mg tablets | dqB6. |
| TOLCAPONE 100mg tablets | dqC1. |
| *TOLCAPONE 200mg tablets | dqC2. |
| TASMAR 100mg tablets | dqC3. |
| *TASMAR 200mg tablets | dqC4. |
| ENTACAPONE 200mg tablets | dqD1. |
| COMTESS 200mg tablets | dqD2. |
| AZILECT 1mg tablets | dqF1. |
| RASAGILINE 1mg tablets | dqFz. |
| NEUPRO 2mg/24hours transdermal patches | dqG1. |
| NEUPRO 4mg/24hours transdermal patches | dqG2. |
| NEUPRO 6mg/24hours transdermal patches | dqG3. |
| NEUPRO 8mg/24hours transdermal patches | dqG4. |
| NEUPRO transdermal patches starter pack | dqG5. |
| NEUPRO 1mg/24hours transdermal patches | dqG6. |
| NEUPRO 3mg/24hours transdermal patches | dqG7. |
| ROTIGOTINE 3mg/24hours transdermal patches | dqGt. |
| ROTIGOTINE 1mg/24hours transdermal patches | dqGu. |
| ROTIGOTINE 2mg+4mg+6mg+8mg/24hours transdermal patches starter pack | dqGv. |
| ROTIGOTINE 8mg/24hours transdermal patches | dqGw. |
| ROTIGOTINE 6mg/24hours transdermal patches | dqGx. |
| ROTIGOTINE 4mg/24hours transdermal patches | dqGy. |
| ROTIGOTINE 2mg/24hours transdermal patches | dqGz. |
| MIRAPEXIN 88micrograms tablets | dqE1. |
| MIRAPEXIN 180micrograms tablets | dqE2. |
| MIRAPEXIN 700micrograms tablets | dqE3. |
| MIRAPEXIN 350micrograms tablets | dqE4. |
| MIRAPEXIN 260micrograms m/r tablets | dqE5. |
| MIRAPEXIN 520micrograms m/r tablets | dqE6. |
| MIRAPEXIN 1.05mg m/r tablets | dqE7. |
| MIRAPEXIN 2.1mg m/r tablets | dqE8. |
| MIRAPEXIN 3.15mg m/r tablets | dqE9. |
| MIRAPEXIN 1.57mg m/r tablets | dqEA. |
| MIRAPEXIN 2.62mg m/r tablets | dqEB. |
| PRAMIPEXOLE 1.1mg tablets | dqEo. |
| PRAMIPEXOLE 2.62mg m/r tablets | dqEp. |
| PRAMIPEXOLE 1.57mg m/r tablets | dqEq. |
| PRAMIPEXOLE 3.15mg m/r tablets | dqEr. |
| PRAMIPEXOLE 2.1mg m/r tablets | dqEs. |
| PRAMIPEXOLE 1.05mg m/r tablets | dqEt. |
| PRAMIPEXOLE 520micrograms m/r tablets | dqEu. |
| PRAMIPEXOLE 260micrograms m/r tablets | dqEv. |
| PRAMIPEXOLE 350micrograms tablets | dqEw. |
| PRAMIPEXOLE 88micrograms tablets | dqEx. |
| PRAMIPEXOLE 180micrograms tablets | dqEy. |
| PRAMIPEXOLE 700micrograms tablets | dqEz. |
