# Supplementary material for: Adult‐onset idiopathic dystonia: A national data‐linkage study to determine epidemiological, social deprivation, and mortality characteristics
Source: Eur J Neurol. 2021 Oct 15;29(1):91–104. doi: 10.1111/ene.15114 (PMC9377012; doi:10.1111/ene.15114)
Supplement: Supplementary file 4 [file ENE-29-91-s002.docx]

**Supplementary Table 4. Levodopa medications used to exclude dystonia patients with co-morbid diagnosis of tremor (Read Code: 1B22.)**

| **Levodopa medication** | **Read code** |
| --- | --- |
| *LEVODOPA 125mg capsules | dq11. |
| *LEVODOPA 250mg capsules | dq12. |
| *LEVODOPA 500mg capsules | dq13. |
| *LEVODOPA 500mg tablets | dq14. |
| *BROCADOPA 125mg capsules | dq15. |
| *BROCADOPA 250mg capsules | dq16. |
| *BROCADOPA 500mg capsules | dq17. |
| *LARODOPA 500mg tablets | dq18. |
| MADOPAR-62.5 capsules | dq21. |
| MADOPAR-125 capsules | dq22. |
| MADOPAR-250 capsules | dq23. |
| MADOPAR-62.5 dispersible tablets | dq24. |
| MADOPAR-125 dispersible tablets | dq25. |
| MADOPAR CR-125 m/r capsules | dq26. |
| CO-BENELDOPA 12.5/50 capsules | dq27. |
| CO-BENELDOPA 25/100 capsules | dq28. |
| CO-BENELDOPA 50/200 capsules | dq29. |
| CO-BENELDOPA 12.5/50 dispersible tablets | dq2a. |
| CO-BENELDOPA 25/100 m/r capsules | dq2b. |
| CO-BENELDOPA 25/100 dispersible tablets | dq2c. |
| SINEMET-110 TAVLETS | dq31. |
| SINEMET-275 tablets | dq32. |
| SINEMET-PLUS tablets | dq33. |
| SINEMET LS tablets | dq34. |
| CO-CARELDOPA 12.5/50 tablets | dq35. |
| CO-CARELDOPA 10/100 tablets | dq36. |
| CO-CARELDOPA 25/100 tablets | dq37. |
| CO-CARELDOPA 25/250 tablets | dq38. |
| SINEMET CR m/r tablets | dq39. |
| HALF-SINEMET CR m/r tablets | dq3A. |
| STALEVO 50mg / 12.5mg / 200mg tablets | dq3B. |
| STALEVO 100mg / 25mg / 200mg tablets | dq3C. |
| STALEVO 150mg /37.5mg / 200mg tablets | dq3D. |
| TILOLEC 100mg/25mg m/r tablets | dq3E. |
| TILOLEC 200mg/50mg m/r tablets | dq3F. |
| DUODOPA 5mg/20mg/mL intestinal gel cassette 100mL | dq3G. |
| CARAMET CR 25mg/100mg m/r tablets | dq3H. |
| CARAMET CR 50mg/200mg m/r tablets | dq3I. |
| STALEVO 200mg/50mg/200mg tablets | dq3J. |
| STALEVO 125mg/31.25mg/200mg tablets | dq3K. |
| STALEVO 75mg/18.75mg/200mg tablets | dq3L. |
| STALEVO 175mg/43.75mg/200mg tablets | dq3M. |
| SASTRAVI 50mg/12.5mg/200mg tablets | dq3N. |
| SASTRAVI 75mg/18.75mg/200mg tablets | dq3O. |
| SASTRAVI 100mg/25mg/200mg tablets | dq3P. |
| SASTRAVI 125mg/31.25mg/200mg tablets | dq3Q. |
| SASTRAVI 150mg/37.5mg/200mg tablets | dq3R. |
| SASTRAVI 175mg/43.75mg/200mg tablets | dq3S. |
| SASTRAVI 200mg/50mg/200mg tablets | dq3T. |
| STANEK 50mg/12.5mg/200mg tablets | dq3U. |
| STANEK 75mg/18.75mg/200mg tablets | dq3V. |
| STANEK 100mg/25mg/200mg tablets | dq3X. |
| STANEK 125mg/31.25mg/200mg tablets | dq3Y. |
| STANEK 150mg/37.5mg/200mg tablets | dq3Z. |
| CO-CARELDOPA 50/200 m/r tablets | dq3a. |
| CO-CARELDOPA 25mg/100mg m/r tablets | dq3b. |
| STANEK 175mg/43.75mg/200mg tablets | dq3c. |
| STANEK 200mg/50mg/200mg tablets | dq3d. |
| LEVODOPA 175mg/CARBIDOPA 43.75mg/ENTACAPONE 200mg tablets | dq3s. |
| LEVODOPA 75mg/CARBIDOPA 18.75mg/ENTACAPONE 200mg tablets | dq3t. |
| LEVODOPA 125mg/CARBIDOPA 31.25mg/ENTACAPONE 200mg tablets | dq3u. |
| LEVODOPA 200mg/CARBIDOPA 50mg/ENTACAPONE 200mg tablets | dq3v. |
| CO-CARELDOPA 5mg/20mg/mL intestinal gel casette 100mL | dq3w. |
| LEVODOPA 150mg / CARBIDOPA 37.5mg / ENTACAPONE 200mg tablets | dq3x. |
| LEVODOPA 100mg / CARBIDOPA 25mg / ENTACAPONE 200mg tablets | dq3y. |
| LEVODOPA 50mg / CARBIDOPA 12.5mg / ENTACAPONE 200mg tablets | dq3z. |
