# Supplementary material for: Adult‐onset idiopathic dystonia: A national data‐linkage study to determine epidemiological, social deprivation, and mortality characteristics
Source: Eur J Neurol. 2021 Oct 15;29(1):91–104. doi: 10.1111/ene.15114 (PMC9377012; doi:10.1111/ene.15114)
Supplement: Supplementary file 5 [file ENE-29-91-s005.docx]

**Supplementary Table 5. Annual incidence and mid-year point prevalence rates for adult-onset idiopathic dystonia cases**

| **Year** | **Number of new dystonia cases** | **Number of new male dystonia cases** | **Number of new female dystonia cases** |  | **General population** | **Incidence per 100,000** | **Mid-year prevalence** | **Male mid-year prevalence** | **Female mid-year prevalence** | **Mid-year general population** | **Mid-year point prevalence per 100,000 (%)** |
| --- | --- | --- | --- | --- | --- | --- | --- | --- | --- | --- | --- |
| 1994 | 829 | 274 | 555 |  | 2,387,092 | 49.85 | 392 | 133 | 259 | 2,387,951 | 16.42 (0.02) |
| 1995 | 875 | 302 | 573 |  | 2,411,453 | 51.13 | 1,263 | 420 | 843 | 2,413,550 | 52.33 (0.05) |
| 1996 | 874 | 296 | 578 |  | 2,426,292 | 52.47 | 2,056 | 689 | 1,367 | 2,429,537 | 84.63 (0.08) |
| 1997 | 996 | 349 | 647 |  | 2,446,118 | 59.20 | 2,950 | 1,010 | 1,940 | 2,450,694 | 120.37 (0.12) |
| 1998 | 1,031 | 387 | 644 |  | 2,456,320 | 59.68 | 3,867 | 1,334 | 2,533 | 2,471,221 | 156.48 (0.16) |
| 1999 | 1,140 | 421 | 719 |  | 2,481,424 | 63.35 | 4,844 | 1,697 | 3,147 | 2,488,703 | 194.64 (0.19) |
| 2000 | 1,182 | 419 | 763 |  | 2,497,982 | 67.01 | 5,796 | 2,033 | 3,763 | 2,506,613 | 231.23 (0.23) |
| 2001 | 1,279 | 435 | 844 |  | 2,515,706 | 71.75 | 6,848 | 2,376 | 4,472 | 2,525,873 | 271.11 (0.27) |
| 2002 | 1,579 | 553 | 1,026 |  | 2,531,727 | 84.92 | 8,055 | 2,795 | 5,260 | 2,543,627 | 316.67 (0.32) |
| 2003 | 1,821 | 635 | 1,186 |  | 2,556,581 | 97.28 | 9,544 | 3,316 | 6,228 | 2,570,531 | 371.29 (0.37) |
| 2004 | 2,029 | 721 | 1,308 |  | 2,589,075 | 104.09 | 11,219 | 3,908 | 7,311 | 2,605,341 | 430.62 (0.43) |
| 2005 | 2,141 | 780 | 1,361 |  | 2,616,458 | 110.42 | 13,007 | 4,540 | 8,467 | 2,635,190 | 493.59 (0.49) |
| 2006 | 2,253 | 843 | 1,410 |  | 2,636,218 | 112.66 | 14,900 | 5,213 | 9,687 | 2,657,535 | 560.67 (0.56) |
| 2007 | 2,212 | 844 | 1,368 |  | 2,650,409 | 109.53 | 16,685 | 5,872 | 10,813 | 2,674,089 | 623.95 (0.62) |
| 2008 | 2,278 | 802 | 1,476 |  | 2,663,164 | 111.37 | 18,490 | 6,522 | 11,968 | 2,689,309 | 687.54 (0.69) |
| 2009 | 2,137 | 792 | 1,345 |  | 2,666,675 | 102.00 | 20,265 | 7,127 | 13,138 | 2,695,097 | 751.92 (0.75) |
| 2010 | 2,112 | 804 | 1,304 |  | 2,667,017 | 100.37 | 21,942 | 7,718 | 14,224 | 2,697,599 | 813.39 (0.81) |
| 2011 | 2,188 | 826 | 1,362 |  | 2,663,147 | 104.39 | 23,523 | 8,251 | 15,272 | 2,695,725 | 872.60 (0.87) |
| 2012 | 2,219 | 845 | 1,374 |  | 2,669,648 | 103.83 | 25,254 | 8,893 | 16,361 | 2,704,405 | 933.81 (0.93) |
| 2013 | 2,048 | 769 | 1,279 |  | 2,670,845 | 98.55 | 26,808 | 9,464 | 17,344 | 2,707,537 | 990.12 (0.99) |
| 2014 | 2,112 | 805 | 1,307 |  | 2,674,547 | 99.68 | 28,303 | 10,050 | 18,253 | 2,713,203 | 1043.16 (1.04) |
| 2015 | 2,035 | 780 | 1,255 |  | 2,674,043 | 93.45 | 29,802 | 10,577 | 19,225 | 2,714,556 | 1097.86 (1.10) |
| 2016 | 2,213 | 844 | 1,369 |  | 2,655,271 | 100.37 | 31,286 | 11,150 | 20,136 | 2,697,528 | 1159.80 (1.16) |
| 2017 | 2,077 | 823 | 1,254 |  | 2,633,845 | 96.21 | 32,662 | 11,701 | 20,961 | 2,677,690 | 1219.78 (1.22) |
| Total | 41,660 | 15,349 | 26,311 |  |  |  |  |  |  |  |  |
